# scGSI: Graph-guided self-supervised integration of paired single-cell multi-omics

**DOI:** 10.1371/journal.pcbi.1014773

**Published:** 2026-09-08

**Authors:** Xiang Chen, Zihan Yang, Xiaoyu Liu, Zhiyi Xie, Wenlu Guo

**Affiliations:** 1 School of Computer Science and Engineering, Hunan University of Science and Technology, Xiangtan, Hunan, China; 2 Hunan Provincial Key Laboratory of Intelligent Computing and Language Information Processing, Hunan University of Science and Technology, Xiangtan, Hunan, China; Wuhan University, CHINA

## Abstract

Paired single-cell multi-omics technologies provide direct within-cell correspondence across molecular layers and offer a powerful route to dissecting cellular heterogeneity and regulatory relationships. However, effective integration requires more than modality mixing: a useful model must accurately align paired cells while preserving modality-specific topological structure and biologically meaningful variation. Existing methods often struggle with topology mismatch across modalities, underuse cross-modal complementarity within paired cells, or improve alignment at the cost of biological fidelity. To address these challenges, we present scGSI, a graph-guided self-supervised framework for paired single-cell multi-omics integration. scGSI combines heterogeneous graph encoders to preserve modality-specific neighborhood structure, a pull-in projection module to stabilize pre-alignment, and a cross-fusion mechanism with contrastive refinement to exploit complementary signals between paired modalities. Across five paired single-cell multi-omics datasets collected from four platforms, scGSI improves paired cell-state alignment while maintaining a favorable balance between modality mixing and biological variation preservation. The learned embeddings also better support downstream analyses, including cell-type discrimination and developmental trajectory inference, showing that accurate alignment need not erase biologically meaningful structure.

## Author summary

Single-cell multi-omics technologies now allow us to measure multiple molecular layers (such as gene expression and chromatin accessibility) within the same cell. However, integrating these different data types into a coherent picture remains challenging because each modality has its own statistical properties and biological meaning. We developed scGSI, a computational framework that uses graph-guided self-supervised learning to align paired single-cell multi-omics data while preserving biologically meaningful variation. scGSI works by first maintaining the unique structure of each data type, then progressively aligning them using paired cellular information, and finally refining the integration through contrastive learning. Across five paired datasets from four experimental platforms, scGSI achieves accurate cross-modal alignment while retaining the biological structure needed for downstream analysis. As a result, the integrated embeddings support clearer cell-type identification and more reliable reconstruction of developmental trajectories. This may help researchers interpret complex cell populations and connect molecular measurements with cell identity and development.

## Introduction

High-throughput single-cell multi-omics sequencing technologies now capture multidimensional genomic landscapes at single-cell resolution, including scRNA-seq [1–3], scATAC-seq [4,5], CITE-seq, and 10x Multiome [6,7]. These platforms delineate molecular portraits of heterogeneous cell populations within tissues. Spatial transcriptomics further complements these molecular profiles by preserving tissue context, and recent graph- and contrastive-learning methods have supported spatial representation learning, histology-based gene-expression prediction, and cross-slice integration and batch correction [8–12]. Although spatially resolved analysis and paired multi-omics integration involve different inputs and analytical objectives, these advances demonstrate the broader value of structure-aware learning across heterogeneous biological measurements. Complementary modalities like DNA methylation and histone modifications [13] further reveal associations in developmental dynamics and regulatory networks [14–16]. Single-cell multi-omics integration links transcriptomes, epigenomes, and proteomes to provide a more complete view of cellular function, tissue development, and disease pathology, thereby advancing precision medicine [17]. However, unimodal approaches offer fragmented perspectives, whereas multi-omics platforms [18,19] face experimental complexity and resource constraints, resulting in relatively scarce paired datasets despite their ability to profile multiple molecular layers within the same cell. These paired datasets are especially valuable because they provide explicit within-cell correspondence across modalities, making them uniquely informative for studying gene regulation and cell-state transitions.

In paired single-cell multi-omics integration, the central goal is not merely to mix modalities in a shared latent space. Instead, an effective method should simultaneously achieve three objectives: accurate cell-to-cell matching across modalities, preservation of modality-specific local structure, and retention of biologically meaningful variation for downstream analysis. These objectives are difficult to satisfy together because paired modalities often differ substantially in sparsity, noise characteristics, and neighborhood geometry.

In the field of single-cell multi-omics data integration, numerous methods have emerged that can be broadly categorized into general frameworks and subclasses tailored specifically for paired data. General frameworks include UnionCom [20], which achieves cell registration via geometric distance matrix matching and projection into shared spaces; scTopoGAN [21], employing topological autoencoders and GANs for latent representation alignment; JointMDS [22], integrating MDS with Wasserstein Procrustes for simultaneous embeddings and correspondence inference; Pamona [23], bridging modality features via graph spectral clustering; MMD-MA [24], optimizing distribution alignment; and SCOT [25], using optimal transport to minimize matching costs. Although these approaches demonstrate efficacy in addressing unpaired scenarios, they frequently rely on global distribution matching and therefore do not fully exploit the direct within-cell correspondence available in paired data. As a result, they may overlook biologically meaningful associations across modalities, causing reduced alignment stability and loss of biological variation during embedding learning [26].

Paired scenarios remain central to single-cell multi-omics integration, in which methods emphasize multi-omics synergy within the same cell. Examples include Mowgli [27], combining non-negative matrix factorization with optimal transport; scMGCL [28], leveraging graph contrastive learning to augment one modality’s graph with another’s for shared representations; MultiVI [29] and scPairing [30] using variational autoencoders to embed into common spaces and generate novel data while capturing multiscale biological structures. However, these paired tools remain limited in three important aspects. First, topology mismatch across modalities is still difficult to address, because methods that force alignment too early can distort local neighborhoods. Second, cross-modal complementarity within paired cells is often underused: for instance, scMGCL leverages graph contrastive learning but does not explicitly model dynamic feature interaction between modalities, limiting robustness in heterogeneous data. Third, alignment may be improved at the expense of biological fidelity: variational approaches such as scPairing and MultiVI are powerful, but their shared latent assumptions can risk oversmoothing biologically relevant variation while also introducing considerable computational cost.

Against this background, growing paired single-cell multi-omics datasets require computational frameworks specifically designed to exploit within-cell correspondence [31]. We therefore present scGSI, a graph-guided self-supervised framework for efficient integration of paired single-cell multi-omics data. The design of scGSI follows a simple principle: preserve modality-specific structure before aggressive alignment, use paired correspondence to guide cross-modal information exchange, and constrain the optimization so that improved alignment does not erase biological variation. Accordingly, scGSI incorporates biological prior information and integrates it through three coordinated components: (i) heterogeneous multi-graph representations that preserve modality-specific topological architectures; (ii) a latent pull-in projection that reduces cross-modal geometric discrepancy before fusion; and (iii) a cross-fusion module with contrastive refinement that explicitly exploits complementary signals between paired modalities. This architecture progressively aligns modalities while retaining the local and biological structure needed for downstream analysis.

This is not a heuristic combination of modules, but a coordinated response to common failure modes. First, excessive early alignment can distort modality-specific neighborhood geometry; this is mitigated by heterogeneous encoders and a pull-in projection that reduce discrepancy before fusion. Second, paired modalities contain complementary information that a shared latent representation may underuse; this is addressed by bidirectional cross-fusion. Third, aggressive matching can oversmooth biologically meaningful variation; this is limited by reconstruction losses that preserve fidelity and by contrastive refinement that enforces paired correspondence without collapsing the representation. Methods such as scPairing employ shared Variational Autoencoders (VAEs) to embed both modalities into a common latent space, which may lead to over-smoothing of minor cell subtypes. In contrast, scGSI adopts separate modal encoders coupled with a bidirectional Transformer cross-fusion mechanism, allowing each modality to preserve its modality-specific structural residuals while attending to complementary signals from its paired counterpart.

Benchmarking across five paired datasets shows that scGSI improves core paired-alignment performance and consistently maintains a strong balance between modality mixing and biological conservation. The resulting embeddings also support clearer cell-type discrimination and more reliable developmental trajectory recovery. scGSI therefore provides a robust and interpretable paradigm for paired single-cell multi-omics integration.

## Materials and methods

### Overview of the scGSI framework

**Fig 1:**
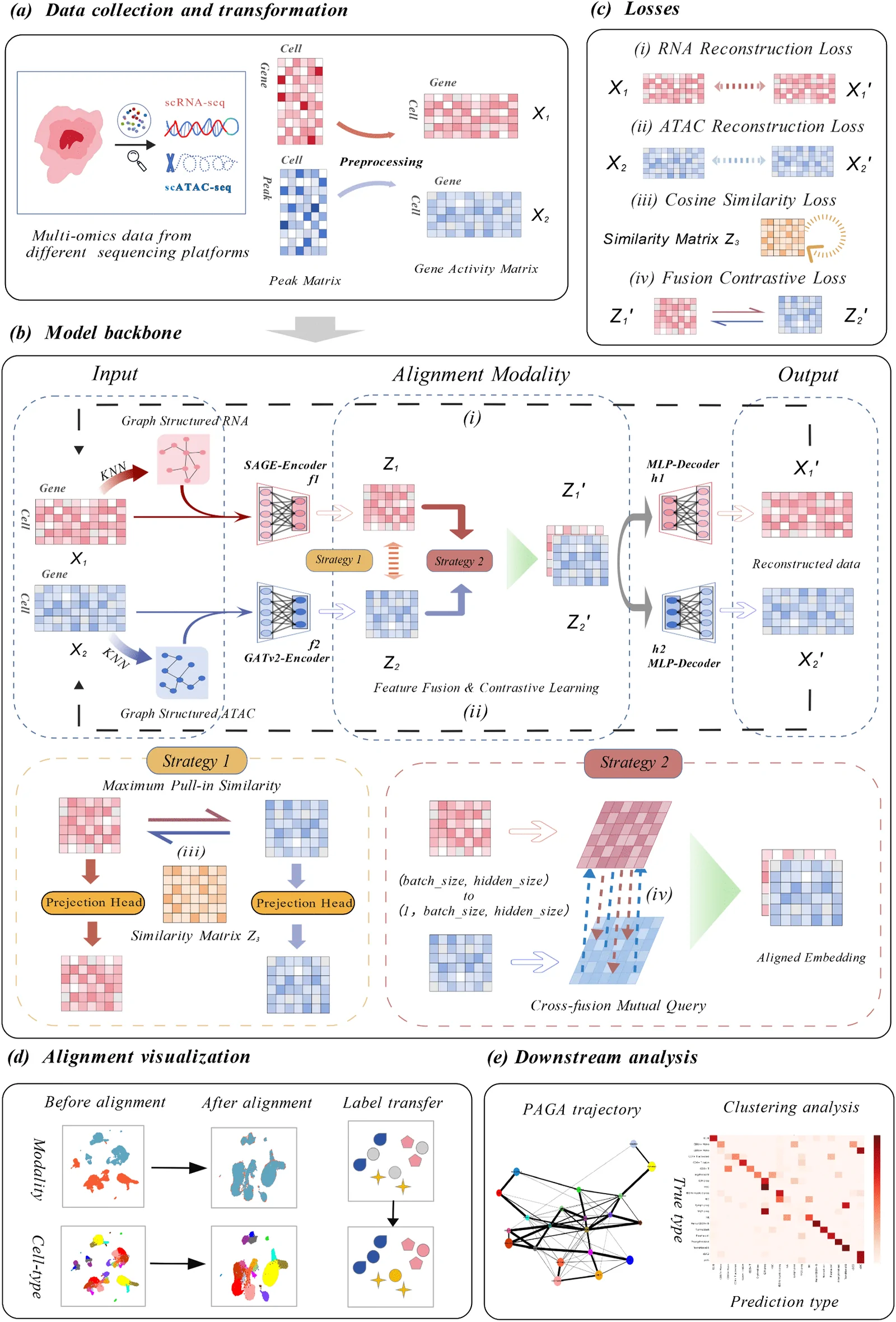
Overview of the scGSI framework for paired single-cell multi-omics integration. scGSI addresses three coupled challenges in paired integration: preserving modality-specific structure, exploiting cross-modal complementarity, and retaining biologically meaningful variation during alignment. **(a)** Biological prior bridging and preprocessing. scATAC-seq peak matrices are converted into gene activity score matrices to establish a biologically informed feature bridge to scRNA-seq. **(b)** Structure-aware progressive alignment. KNN graphs preserve local topology, GraphSAGE and GATv2 extract modality-specific latent features, the pull-in projection reduces initial cross-modal discrepancy, and bidirectional cross-fusion exchanges complementary paired signals. **(c)** Structure-preserving self-supervised objectives. RNA and ATAC reconstruction losses preserve modality-specific fidelity, cosine similarity promotes paired consistency, and fusion contrastive loss strengthens true paired correspondences against non-paired cells. **(d)** Alignment outcome. The learned embedding space improves paired cell-state matching while maintaining effective modality mixing. **(e)** Downstream biological utility. The aligned embeddings support trajectory inference and cell-state discrimination.

scGSI is a graph-guided self-supervised framework designed for paired single-cell multi-omics integration (Fig 1). Its design follows three principles introduced in the Introduction: preserve modality-specific structure before aggressive alignment, use paired correspondence to guide cross-modal information exchange, and constrain optimization so that improved alignment does not erase biological variation. Accordingly, the framework consists of four stages: (i) biological prior bridging and graph construction, which establish comparable inputs while retaining modality-specific local topology; (ii) modality-specific graph encoding, which extracts latent representations adapted to the distinct statistical properties of each modality; (iii) progressive cross-modal alignment, which first reduces geometric discrepancy and then performs paired feature fusion; and (iv) self-supervised structure-preserving optimization, which balances alignment pressure against faithful reconstruction of the original modalities.

### Data collection and preprocessing

We selected five paired multi-omics datasets from the GEO database and the published literature (see Table A in S1 Appendix). These datasets originated from four major platforms: 10x Multiome, CITE-seq, SNARE-seq, and TEA-seq, ensuring cross-technology validation of the integration framework.

To enable effective alignment between scATAC-seq and scRNA-seq, we transformed the non-RNA modality into a representation that shares the feature space with scRNA-seq. This step is not merely a convenience for preprocessing; rather, it provides a biologically informed bridge that makes cross-modal comparison possible while retaining regulatory information derived from chromatin accessibility. Existing methods (such as ArchR [32], Cicero [33], scATAC-pro [34], and Signac [35]) differ primarily in the computation and weighting mechanisms for gene region accessibility. We used Signac [35] throughout to standardize this transformation step (see Section 1.1 in S1 Appendix), and then applied separate preprocessing pipelines to scRNA-seq and transformed scATAC-seq data (see Section 1.2 in S1 Appendix).

### Problem formulation and overview

Let $X^{\left(r\right)}\in {\mathbb{R}}^{N\times D_{r}}$ and $X^{\left(a\right)}\in {\mathbb{R}}^{N\times D_{a}}$ denote the preprocessed input matrices for RNA and ATAC modalities, respectively, where *N* is the number of paired cells and $D_{r}$ and $D_{a}$ are the corresponding feature dimensions. The goal of scGSI is to learn a shared low-dimensional representation in which the two modalities of the same cell are accurately aligned, while the local structure and biologically meaningful variation of each modality are preserved.

The main challenge is that paired modalities are not statistically interchangeable: scRNA-seq is comparatively dense and smooth, whereas scATAC-seq is sparse, noisy, and highly heterogeneous. Directly forcing both modalities into the same latent space can therefore distort neighborhood geometry before the model has extracted stable modality-specific structure. To avoid this failure mode, scGSI first encodes each modality with a dedicated graph-based encoder, then performs progressive pre-alignment and cross-modal fusion, and finally applies reconstruction and contrastive objectives to preserve both alignment accuracy and biological fidelity.

### Modality-specific graph encoding

Data were partitioned into batches *B* = 256 as $\{X_{b}^{\left(r\right)}{\}}_{b=1}^{M}$ and $\{X_{b}^{\left(a\right)}{\}}_{b=1}^{M}$ (M=⌈N/B⌉). For batch *b*, the RNA modality latent embedding $Z_{enc,b}^{\left(r\right)}\in {\mathbb{R}}^{B\times L}$ (*L* is the latent dimension) is computed using the GraphSAGE encoder as:


$$\begin{array}{c} Z_{enc,b}^{\left(r\right)}=E_{SAGE}^{\left(r\right)}(X_{b}^{\left(r\right)},G_{b}^{\left(r\right)}) \end{array}$$ (1)

where $G_{b}^{\left(r\right)}=({𝒱}_{b}^{\left(r\right)},{ℰ}_{b}^{\left(r\right)})$ is the batch local neighborhood graph; see Section 1.3 in S1 Appendix.

Similarly, the ATAC modality embedding $Z_{enc,b}^{\left(a\right)}\in {\mathbb{R}}^{B\times L}$ is computed by the GATv2 encoder:


$$\begin{array}{c} Z_{enc,b}^{\left(a\right)}=E_{GATv2}^{\left(a\right)}(X_{b}^{\left(a\right)},G_{b}^{\left(a\right)}) \end{array}$$ (2)

Concatenating the batch-level outputs yields $Z_{enc}^{\left(r\right)},Z_{enc}^{\left(a\right)}\in {\mathbb{R}}^{N\times L}$, which serve as the inputs to the pre-alignment module.

These encoder mappings are implemented with dedicated modality-specific graph neural network (GNN) variants rather than a shared encoder, thereby preserving local topology before cross-modal alignment. Because the two modalities encode distinct biological signals and have different noise patterns, they benefit from different inductive biases. scRNA-seq data are relatively dense and smooth, so neighborhood mean aggregation through GraphSAGE can stably extract local features while preserving expression continuity. By contrast, scATAC-seq data are sparser, noisier, and more heterogeneous; fixed aggregation is therefore less adequate for identifying informative regulatory neighborhoods, and adaptive attention is better suited for emphasizing biologically relevant interactions. We therefore adopt GATv2 for the ATAC modality, using adaptive attention to balance the contribution of different neighbors and to mine deeper regulatory associations from noisy open-chromatin signals. This choice is not intended as a universal requirement of graph learning, but as a modality-aware design that is empirically supported by the analyses in Fig O in S1 Appendix, where we further compare encoder combinations under objectives including GC and LAT.

The GraphSAGE encoder extracts smooth latent representations from the gene expression matrix $X^{\left(r\right)}\in {\mathbb{R}}^{N\times D_{r}}$ ($D_{r}$ is the gene dimension) through iterative neighborhood aggregation. The hidden representation $h_{v}^{\left(l\right)}\in {\mathbb{R}}^{h}$ (*h* is the hidden dimension) of node *v* at layer *l* is computed as:


$$\begin{array}{c} h_{v}^{\left(l+1\right)}=\sigma \left(W^{\left(l\right)}\cdot \text{AGG}\left(\{h_{u}^{\left(l\right)}|u\in 𝒩(v)\cup \{v\}\}\right)+b^{\left(l\right)}\right) \end{array}$$ (3)

where AGG(·) is the aggregation function, 𝒩(v) is the set of neighbors of *v*, *W*^(*l*)^ and *b*^(*l*)^ are learnable parameters, σ is ReLU activation, and the initial layer $h_{v}^{\left(0\right)}=x_{v}^{\left(r\right)}$ (node features of *v*).

The GATv2 encoder extracts weighted latent representations from the gene activity score matrix $X^{\left(a\right)}\in {\mathbb{R}}^{N\times D_{a}}$ ($D_{a}$ is the gene activity dimension) using multi-head attention. The hidden representation $h_{v}^{\left(l+1\right)}\in {\mathbb{R}}^{H}$ of node *v* at layer *l* is computed through *H*-head attention as:


$$\begin{array}{c} h_{v}^{\left(l+1\right)}={\|}_{i=1}^{H}\sigma \left(\sum_{u\in 𝒩(v)\cup \{v\}}\alpha_{vu}^{\left(i\right)}W^{\left(i\right)}h_{u}^{\left(l\right)}\right) \end{array}$$ (4)

where ‖ denotes head-wise concatenation along the feature dimension, $\alpha_{vu}^{\left(i\right)}$ is the attention coefficient, and the initial $h_{v}^{\left(0\right)}=x_{v}^{\left(a\right)}$.

This stage yields modality-specific latent representations that retain local graph structure before any cross-modal interaction is imposed.

### Progressive cross-modal alignment

#### Pre-align projection.

The similarity projection module addresses the topology mismatch between modalities by providing a soft pre-alignment before full fusion. Its purpose is not to force the final integrated representation directly, but to reduce the initial geometric discrepancy between RNA and ATAC latent spaces so that subsequent cross-modal interaction becomes more stable. It consists of two modality-specific projection heads, each defined as a two-layer MLP and applied row-wise to the corresponding encoder output:


$$\begin{array}{c} f_{\phi }^{\left(m\right)}(z)=W_{2}^{\left(m\right)}\mathrm{ReLU}(W_{1}^{\left(m\right)}z),\;m\in \{r,a\}, \end{array}$$ (5)

where $z\in {\mathbb{R}}^{L}$, $W_{1}^{\left(m\right)}\in {\mathbb{R}}^{h_{p}\times L}$, $W_{2}^{\left(m\right)}\in {\mathbb{R}}^{L\times h_{p}}$, and $h_{p}$ is the projection-head hidden dimension.

For the low-dimensional encoder outputs $Z_{enc}^{\left(r\right)}$ and $Z_{enc}^{\left(a\right)}$, the module produces projected representations with the same dimensionality *L*:


$$\begin{array}{c} Z_{proj}^{\left(r\right)}=f_{\phi }^{\left(r\right)}(Z_{enc}^{\left(r\right)}),\;Z_{proj}^{\left(a\right)}=f_{\phi }^{\left(a\right)}(Z_{enc}^{\left(a\right)}), \end{array}$$ (6)

where $Z_{proj}^{\left(r\right)},Z_{proj}^{\left(a\right)}\in {\mathbb{R}}^{N\times L}$ are used explicitly as the inputs to the subsequent cross-modality fusion module.

For modalities with non-overlapping feature spaces (e.g., CITE-seq protein counts), each modality is first encoded into its own latent space; modality-specific projection heads then map these latents into a common space before fusion, without requiring shared feature names.

#### Cross-modality fusion.

Paired cells contain complementary but non-identical information across modalities. To explicitly exploit this complementarity, we designed a Transformer-style multi-head attention architecture that allows each modality to selectively query informative signals from its paired counterpart rather than merely concatenating features or enforcing a shared encoder. The inputs are the RNA and ATAC projected embeddings $Z_{proj}^{\left(r\right)},Z_{proj}^{\left(a\right)}\in {\mathbb{R}}^{N\times L}$, expanded to (1, *N*, *L*) to be compatible with the batch-first format.

For the RNA-to-ATAC direction, the attention output $A_{h}^{\left(r\to a\right)}\in {\mathbb{R}}^{N\times d_{h}}$ ($d_{h}=L/H$, where *H* is the number of heads) for head *h* is computed as:


$$\begin{array}{c} A_{h}^{\left(r\to a\right)}=\text{softmax}\left(\frac{Q_{h}^{\left(a\right)}(K_{h}^{\left(r\right)}{)}^{T}}{\sqrt{d_{h}}}\right)V_{h}^{\left(r\right)} \end{array}$$ (7)

where query $Q_{h}^{\left(a\right)}=Z_{proj}^{\left(a\right)}W_{Q}^{\left(h\right)}$, key $K_{h}^{\left(r\right)}=Z_{proj}^{\left(r\right)}W_{K}^{\left(h\right)}$, and value $V_{h}^{\left(r\right)}=Z_{proj}^{\left(r\right)}W_{V}^{\left(h\right)}$, with $W_{Q}^{\left(h\right)},W_{K}^{\left(h\right)},W_{V}^{\left(h\right)}\in {\mathbb{R}}^{L\times d_{h}}$ as learnable matrices; division by $\sqrt{d_{h}}$ stabilizes the attention logits. After multi-head concatenation, $A^{\left(r\to a\right)}=[A_{1}^{\left(r\to a\right)};...;A_{H}^{\left(r\to a\right)}]W_{O}$ ($W_{O}$ is the output projection). The ATAC fused embedding $Z_{fuse}^{\left(a\right)}$ is updated through residual LayerNorm and FFN as:


$$\begin{array}{c} H_{fuse}^{\left(a\right)}=\text{LayerNorm}(Z_{proj}^{\left(a\right)}+A^{\left(r\to a\right)}) \end{array}$$ (8)

$$\begin{array}{c} Z_{fuse}^{\left(a\right)}=\text{LayerNorm}(H_{fuse}^{\left(a\right)}+\text{FFN}(H_{fuse}^{\left(a\right)})) \end{array}$$ (9)

Similarly, the ATAC-to-RNA direction uses $Z_{proj}^{\left(r\right)}$ as the query and $Z_{proj}^{\left(a\right)}$ as the key and value to compute $A^{\left(a\to r\right)}$, generating $Z_{fuse}^{\left(r\right)}$ through the same residual LayerNorm and FFN operations. By using bidirectional fusion, scGSI allows each modality to refine its representation using paired complementary context while retaining its own residual identity.

### Self-supervised structure-preserving optimization

After cross-modal fusion, the model is optimized with reconstruction and alignment objectives that serve distinct and competing roles. Reconstruction losses preserve modality-specific information and discourage biologically implausible collapse by requiring the fused embedding to retain sufficient information to reconstruct each modality’s original high-dimensional features. Alignment losses (cosine and contrastive), by contrast, improve correspondence between paired cells. Crucially, the reconstruction terms act on the same fused embedding as the contrastive term, creating a direct gradient conflict: if alignment pressure were to erase modality-specific structure, reconstruction quality would degrade accordingly. The cosine loss is applied to the modality-specific encoder outputs before projection and fusion, providing an early soft alignment constraint rather than forcing strong consistency on the final fused representation. Together, these competing objectives limit over-alignment and preserve structure that remains useful for downstream biological analysis.

#### MLP as decoder.

Inspired by stAI [36], we used two independent multilayer perceptron (MLP) decoders rather than graph-based decoders for modality-specific reconstruction. MLPs have lower computational complexity (O(N·L·D)) than the graph aggregation layers in the encoders (O(E·h)), which keeps the decoder lightweight and helps stabilize optimization. Through nonlinear mapping, they also reconstruct the original high-dimensional features effectively under self-supervised reconstruction loss. For modality m∈{r,a} and cell *i*, let $z_{fuse,i}^{\left(m\right)}\in {\mathbb{R}}^{L}$ denote the corresponding row of $Z_{fuse}^{\left(m\right)}$. The first-layer hidden representation $h_{i}^{\left(m\right)}\in {\mathbb{R}}^{h}$ (*h* is the hidden dimension) is computed as:


$$\begin{array}{c} h_{i}^{\left(m\right)}=\sigma (W_{1}^{\left(m\right)}z_{fuse,i}^{\left(m\right)}+b_{1}^{\left(m\right)}) \end{array}$$ (10)

where $W_{1}^{\left(m\right)}\in {\mathbb{R}}^{h\times L}$ and $b_{1}^{\left(m\right)}\in {\mathbb{R}}^{h}$ are learnable parameters, σ=ReLU, followed by dropout: $h_{i}^{(m)\prime }=\text{Dropout}(h_{i}^{\left(m\right)},p)$. The reconstructed feature vector is:


$$\begin{array}{c} {\hat{x}}_{i}^{\left(m\right)}=W_{2}^{\left(m\right)}h_{i}^{(m)\prime }+b_{2}^{\left(m\right)} \end{array}$$ (11)

where $W_{2}^{\left(m\right)}\in {\mathbb{R}}^{D_{m}\times h}$ and $b_{2}^{\left(m\right)}\in {\mathbb{R}}^{D_{m}}$. Thus, the modality-specific decoder is:


$$\begin{array}{c} f_{dec}^{\left(m\right)}(z_{fuse,i}^{\left(m\right)})=W_{2}^{\left(m\right)}\cdot \text{Dropout}(\sigma (W_{1}^{\left(m\right)}z_{fuse,i}^{\left(m\right)}+b_{1}^{\left(m\right)}),p)+b_{2}^{\left(m\right)} \end{array}$$ (12)

with parameters initialized from a normal distribution to stabilize training dynamics.

#### Reconstruction loss.

We used weighted mean squared error (MSE) to measure the consistency between the gene expression matrix reconstructed from latent embeddings and the original input, supplemented with L2 regularization to prevent overfitting. For input $X^{\left(r\right)}\in {\mathbb{R}}^{N\times D_{r}}$ and reconstruction ${\hat{X}}^{\left(r\right)}\in {\mathbb{R}}^{N\times D_{r}}$, RNA reconstruction loss is defined as:


$$\begin{array}{c} {ℒ}_{\text{recon\_rna}}=\omega_{r}\cdot \frac{1}{ND_{r}}\sum_{i=1}^{N}\sum_{j=1}^{D_{r}}(X_{ij}^{\left(r\right)}-{\hat{X}}_{ij}^{\left(r\right)}{)}^{2}+\lambda_{r}\sum_{\theta \in \Theta_{\text{enc}}}\|\theta {\|}_{2} \end{array}$$ (13)

where $\omega_{r}$ is the positive sample (non-zero expression positions) weight, $\lambda_{r}$ is the L2 regularization coefficient, and $\Theta_{\text{enc}}$ denotes the GraphSAGE encoder parameters. Similarly, ATAC reconstruction loss is defined as:


$$\begin{array}{c} {ℒ}_{\text{recon\_atac}}=\omega_{a}\cdot \frac{1}{ND_{a}}\sum_{i=1}^{N}\sum_{j=1}^{D_{a}}(X_{ij}^{\left(a\right)}-{\hat{X}}_{ij}^{\left(a\right)}{)}^{2}+\lambda_{a}\sum_{\theta \in \Theta_{\text{enc}}}\|\theta {\|}_{2} \end{array}$$ (14)

where $\Theta_{\text{enc}}$ are the GATv2 encoder parameters.

#### Cosine alignment loss.

This loss minimizes directional deviation between the modality-specific encoder outputs $Z_{enc}^{\left(r\right)},Z_{enc}^{\left(a\right)}\in {\mathbb{R}}^{N\times L}$, providing an early soft alignment constraint before projection and cross-fusion. The loss is:


$$\begin{array}{c} {ℒ}_{\text{cos}}=-\frac{1}{N}\sum_{i=1}^{N}\frac{Z_{enc,i}^{\left(r\right)}\cdot Z_{enc,i}^{\left(a\right)}}{\|Z_{enc,i}^{\left(r\right)}{\|}_{2}\|Z_{enc,i}^{\left(a\right)}{\|}_{2}}\cdot \tau^{-1} \end{array}$$ (15)

where τ is the temperature parameter and $Z_{enc,i}^{\left(m\right)}$ is the modality-specific encoder output of cell *i* in modality *m*.

#### Contrastive loss.

We employ the InfoNCE contrastive paradigm for fused embeddings $Z_{fuse}^{\left(r\right)},Z_{fuse}^{\left(a\right)}\in {\mathbb{R}}^{N\times L}$. This term is the main discriminative force that explicitly strengthens true paired correspondences while pushing apart embeddings from different cells:


$$\begin{array}{c} {ℒ}_{\text{contrast}}=-\frac{1}{N}\sum_{i=1}^{N}\log\frac{\exp(\text{sim}(Z_{fuse,i}^{\left(r\right)},Z_{fuse,i}^{\left(a\right)})/\tau )}{\sum_{j=1}^{N}\exp(\text{sim}(Z_{fuse,i}^{\left(r\right)},Z_{fuse,j}^{\left(a\right)})/\tau )} \end{array}$$ (16)

where $\text{sim}(u,v)=u^{T}v/(\|u{\|}_{2}\|v{\|}_{2})$ is cosine similarity.

#### Overall objective.

The total loss function is the weighted sum of the above losses:


$$\begin{array}{c} {ℒ}_{\text{total}}=w_{1}{ℒ}_{\text{recon\_rna}}+w_{2}{ℒ}_{\text{recon\_atac}}+w_{3}{ℒ}_{\text{cos}}+w_{4}{ℒ}_{\text{contrast}} \end{array}$$ (17)

where $w_{i}$ are initial weights. In this formulation, reconstruction losses preserve modality-specific fidelity, the cosine term encourages paired consistency at the latent level, and the contrastive term sharpens cell-level discrimination after fusion.

### Evaluation metrics

Following existing studies [20,24] and benchmark evaluations [37], we grouped the evaluation criteria according to the biological and computational claims of the model. FOSCTTM and LAT were used as core metrics for paired cell-state matching. ARI, NMI and AMI were used to quantify cell-type prediction accuracy, and clustering resolution was evaluated based on the accuracy of the K-means algorithm. We measured fusion uniformity between modalities through the Omics Mixing Index (OMI) and introduced the Biological Conservation Index (BCI) to evaluate label-based conservation of cell-type organization in the integrated embedding, which directly reflects whether cell-type structure remains coherent after integration. Trajectory consistency was primarily assessed using F1-score and Recall, with complementary support from permutation testing for statistical evaluation. Together, these metrics cover alignment accuracy, downstream discriminative utility, modality mixing, and biological conservation. For more details, please refer to Section 1.4 in S1 Appendix.

### Benchmarking and parameter configuration

We selected nine methods for systematic comparison, covering general integration frameworks and dedicated methods for paired data: UnionCom [20], scTopoGAN [21], JointMDS [22], Pamona [23], MMD-MA [24], SCOT [25], scMGCL [28], MultiVI [29] and scPairing [30]. Detailed explanations are provided in Section 1.5 in S1 Appendix. These methods represent mainstream technical paradigms in the current field of multi-omics integration.

All algorithms were implemented in Python 3.12 and PyTorch (version 2.4.0 + cu118). With FOSCTTM as the optimization objective, the default hyperparameters were fixed across all datasets after initial grid search optimization on a representative dataset. As described in Fig 6D–6E and Figs M–N in S1 Appendix, scGSI requires no per-dataset hyperparameter tuning. To ensure fairness and repeatability of the evaluation, all methods were evaluated under a uniform nearest-neighbor parameter (*k*), and the preset number of clusters in cluster analysis was determined based on the actual number of cell types in the dataset. The experimental procedures and the setting of hyperparameter values of each baseline method followed the author-recommended defaults in the original literature and code repositories, without an additional per-method grid search. More detailed information is provided in Tables B–C in S1 Appendix. End-to-end runtime and peak memory usage were measured for all methods on each dataset under identical hardware and software conditions. Across datasets, scGSI shows moderate runtime but the lowest peak memory among all benchmarked methods (Fig S in S1 Appendix).

## Results

### scGSI improves paired cross-modal cell alignment

BMMC provides a stringent test of paired cross-modal alignment because it contains closely related immune populations with subtle transcriptional differences. The central question on this dataset is therefore simple: can a method bring true RNA-ATAC pairs together without collapsing neighboring immune states into a single mixed manifold?

**Fig 2:**
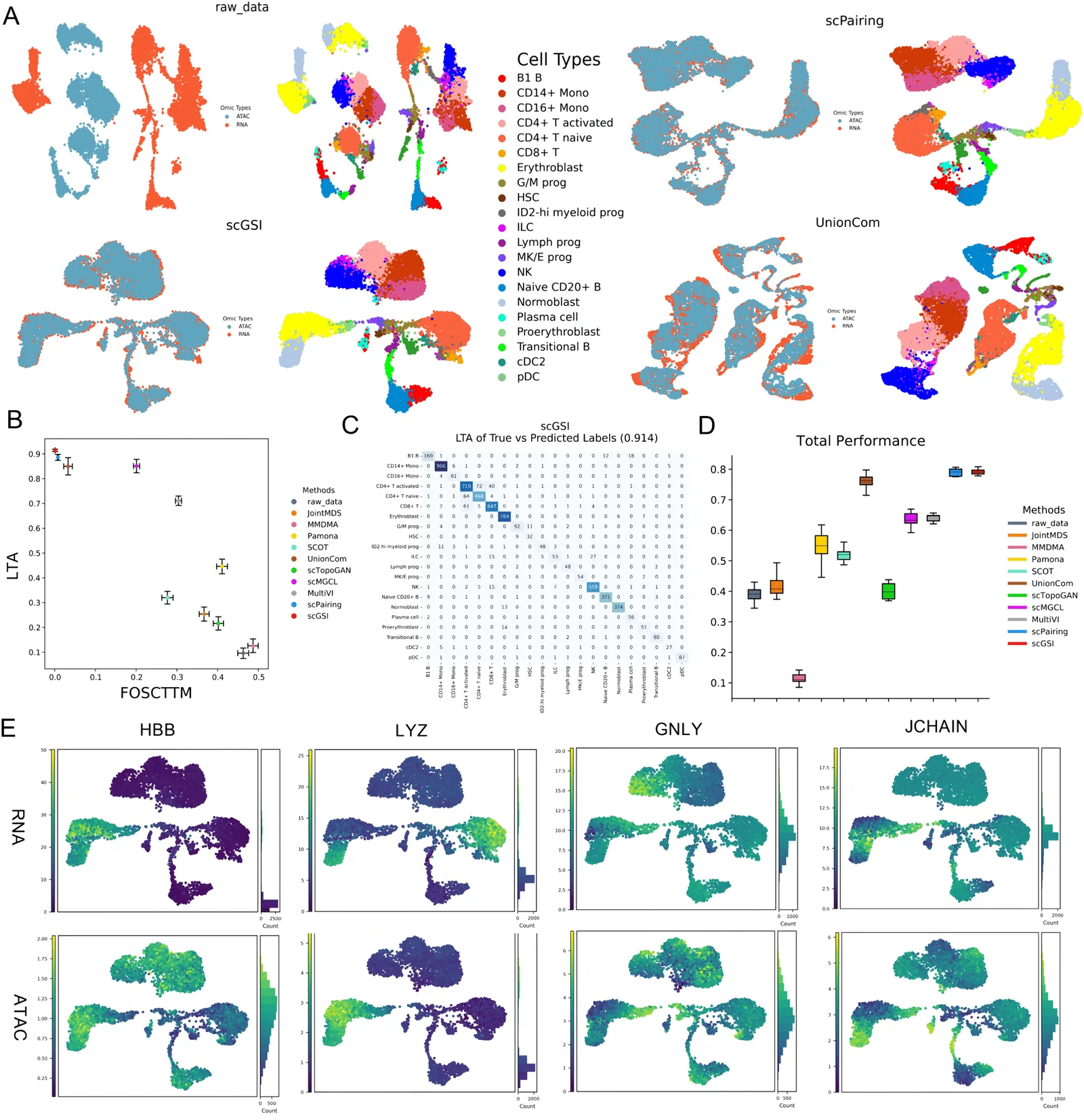
scGSI improves paired cross-modal cell alignment on the BMMC dataset. Assessment of paired cell matching across modalities while preserving separation among closely related immune subpopulations. **(A)** UMAP visualization before and after integration, colored by modality (left) and cell type (right). **(B)** Joint scatter comparison of LAT and FOSCTTM, highlighting paired cell-matching performance across methods. Each point represents the mean performance, and error bars denote the standard deviation across multiple runs, with smaller error bars indicating more stable performance. **(C)** ATAC-to-RNA label transfer confusion matrix, showing the remaining ambiguity among closely related T-cell subpopulations. **(D)** Box plot of overall performance scores, where Performance = $\frac{1}{7}(\text{LAT}+\text{ARI}+\text{NMI}+\text{AMI}+\text{OMI}+\text{BCI}+\text{Accuracy})$. **(E)** Marker-gene expression (HBB, LYZ, GNLY, JCHAIN) levels in ATAC cells and RNA cells after integration.

Before integration, RNA and ATAC occupy separated regions of the embedding space, and several related T-cell populations show weak boundaries (Fig 2A). After integration, scGSI produces the clearest paired structure among the leading methods: the two modalities mix effectively, cells of the same annotated type co-localize, and nearby immune states remain visibly distinguishable. The full UMAP comparison across methods is provided in Fig A in S1 Appendix.

In the joint LAT-FOSCTTM scatter comparison (Fig 2B), scGSI achieves the strongest and most concentrated distribution on both metrics, showing that true cross-modal counterparts are brought closer together while incorrect matches remain farther apart. Several competing methods perform well on one alignment metric but not the other, whereas scGSI remains strong on both pairwise matching accuracy and label transfer.

The remaining errors are concentrated in biologically adjacent T-cell states. The confusion matrix in Fig 2C and the full comparison in Fig B in S1 Appendix show that most ambiguity occurs among CD4 + T activated, CD4 + T naive, and CD8 + T cells. This residual confusion is biologically plausible because these states share core transcriptional programs, and activated CD4 + T cells can transiently resemble naive-like states [38]. Even so, scGSI reduces these ambiguities more effectively than the competing methods while preserving the broader immune landscape. At the dataset level, the summary box plots in Fig 2D and Fig P in S1 Appendix place scGSI at the top of the benchmark, ahead of scPairing and UnionCom.

The alignment effectiveness of scGSI was further confirmed through biological verification of marker genes [5,19]. Fig 2E reveals the heterogeneity of transcriptome and epigenome expression and their regulatory relationship at the single-cell level: (i) As a core marker gene of the Erythroid lineage [39], HBB is highly enriched in erythroid differentiation branches, showing strong mRNA expression. Meanwhile, this locus remains open at the epigenetic level, confirming the synergistic regulatory mechanism of “epigenetic openness driving transcriptional activation” [40,41]. (ii) LYZ, a classic monocyte marker, shows high mRNA expression in the CD14+ and CD16 + monocyte subsets, whereas chromatin accessibility at the corresponding sites is relatively low. Significant epigenetic activation at this site was observed in normoblasts, demonstrating the phenomenon of “epigenetic-transcriptional decoupling” [4]. (iii) The classic marker gene GNLY of natural killer cells (NK cells) has a specific epigenetic open conformation that plays an epigenetic priming role, which helps drive the differentiation of NK cells into mature functional immune subsets [4]. (iv) JCHAIN precisely identifies terminally differentiated Plasma cells, where the locus shows marked nucleosome depletion and a highly accessible state. This deep epigenetic remodeling is consistent with terminal commitment and limited developmental plasticity [42]. It also highlights the strong coupling of chromatin openness and transcription in specialized cells.

Overall, the BMMC results indicate that scGSI improves paired cell-to-cell alignment while preserving the local regulatory patterns visible across modalities.

### scGSI balances modality mixing with biological variation preservation

Accurate integration is not enough if the shared embedding is obtained by washing out meaningful biology. We therefore used the CITE_PBMC dataset to test the most important tradeoff in paired integration: whether stronger modality mixing can be achieved without erasing cell-state structure.

**Fig 3:**
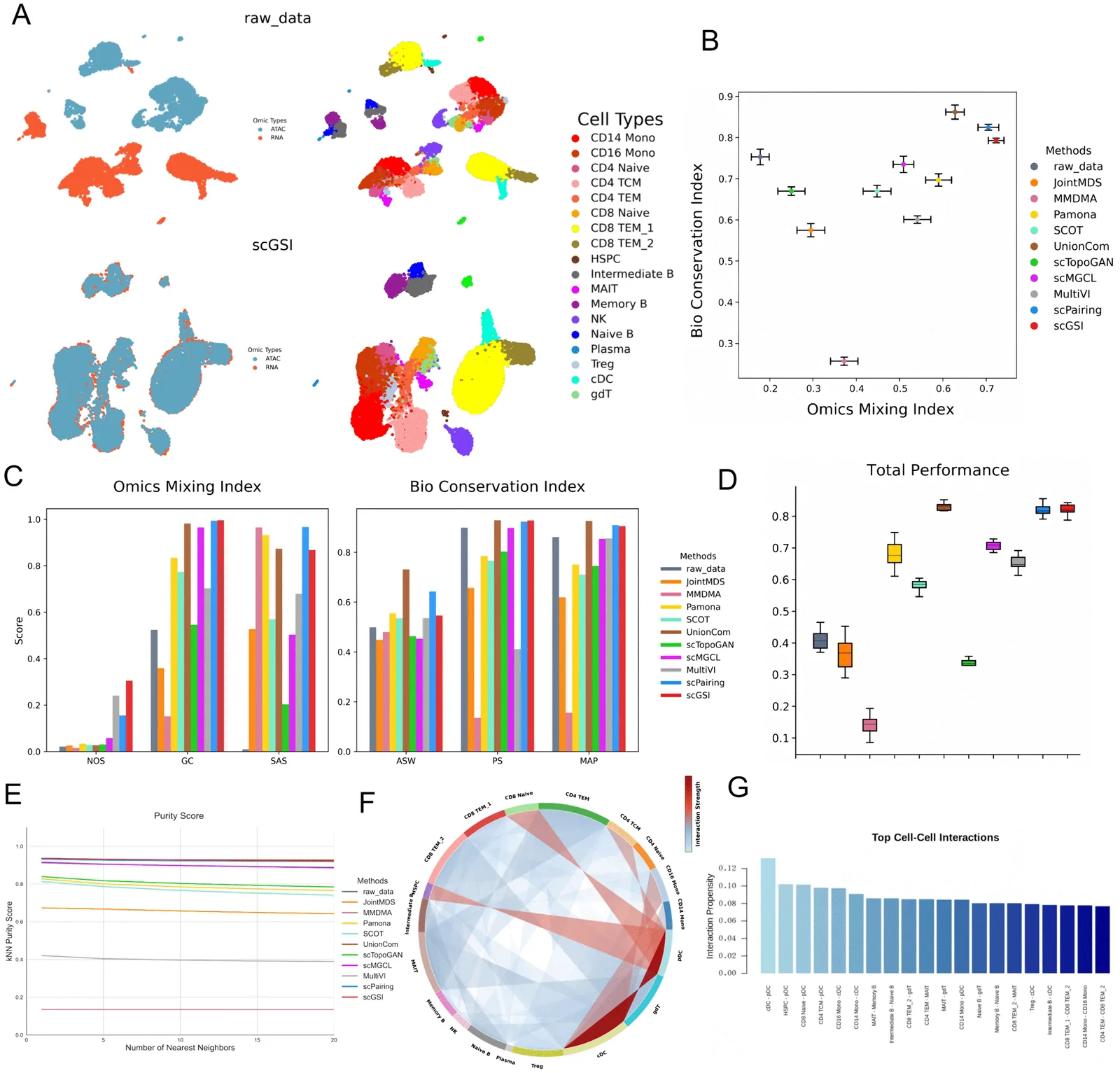
scGSI balances modality mixing with biological variation preservation on the CITE_PBMC dataset. Assessment of the main integration tradeoff: stronger cross-modal alignment without loss of meaningful cell-state structure. **(A)** UMAP visualization before and after integration, colored by modality (left) and cell type (right). **(B)** Joint scatter comparison of omics mixing and biological variation preservation, highlighting the balance achieved by scGSI. **(C)** Breakdown of individual metrics within modality mixing and biological conservation, revealing which aspects of the tradeoff are best maintained. **(D)** Box plot of overall performance scores. **(E)** Purity score sensitivity analysis across neighborhood sizes, assessing the stability of biological conservation in the integrated embedding. **(F)** Chord diagrams of potential global cell-cell interactions inferred from aligned cell embeddings, where arc thickness and color intensity represent interaction tendency. **(G)** Bar chart of the top 20 cell-type interactions, quantifying the highest-ranked pairs.

scGSI performs well on both sides of this tradeoff. The UMAP patterns in Fig 3A and Fig D in S1 Appendix are consistent with the quantitative summary in Fig P in S1 Appendix and Fig 3D: scGSI achieves the lowest FOSCTTM and the highest label transfer accuracy, indicating strong cross-modal matching. At the same time, the joint scatter comparison in Fig 3B shows that scGSI also maintains one of the most favorable balances between omics mixing and biological conservation. It achieves the highest omics-mixing score (0.723) while ranking competitively on cell-variation preservation (0.793), behind only UnionCom and scPairing. This combination suggests that the gain is not driven by simple over-mixing.

The metric breakdown clarifies where this advantage comes from (Fig 3C). Within the omics mixing index (OMI), scGSI attains the highest Neighborhood Overlap Score (NOS = 0.305) and Graph Connectivity (GC = 0.996), indicating stronger local cross-modal consistency and tighter within-type connectivity. Although scPairing achieves the highest Seurat Alignment Score (SAS = 0.967), scGSI remains competitive on the biological conservation index (BCI), including Purity Score (PS) and Mean Average Precision (MAP), indicating that cell-type organization in the integrated embedding remains coherent. UnionCom performs best on ASW, but this comes with weaker alignment performance overall.

The purity-sensitivity analysis in Fig 3E further shows that scGSI is stable across neighborhood sizes, with performance comparable to scPairing and UnionCom and substantially better than MMD-MA. The overall performance box plot (Fig 3D) shows that scGSI ranks first and is stable overall (comparable with scPairing) while maintaining one of the clearest balances between integration strength and biological fidelity.

To characterize potential interactions among peripheral blood mononuclear cell types, we constructed a cell-cell similarity network based on aligned cell embeddings (Fig 3F–3G), and the chord diagram summarizes interaction propensity at different intensity levels [43]. Specifically, we define interaction propensity as the average cosine similarity between cell-type centroids in the aligned embedding space, averaged only over centroid pairs whose similarity exceeds a given intensity threshold, serving as a proxy for cell-cell interaction tendency rather than direct ligand-receptor communication. The strongest interactions involve plasmacytoid dendritic cells (pDC) with classical dendritic cells (cDC), hematopoietic stem and progenitor cells (HSPC), and T-cell subsets (CD8 Naive and CD4 TCM), which may reflect coordinated antigen presentation by dendritic-cell subsets [44] as well as coupling between early hematopoietic development and T-cell immune activation. Moderate interactions are observed between monocytes (CD14 Mono and CD16 Mono) and cDC, and between mucosal-associated invariant T cells (MAIT) and Memory B cells, highlighting developmental continuity within the myeloid lineage and functional crosstalk between unconventional T cells and the humoral immune system [45]. Interactions within and between CD4/CD8 T-cell subsets are comparatively weak [5], but they still maintain a clear lineage-specific pattern.

The bar-chart analysis reaches the same conclusion and places pDC and cDC at the center of the interaction network, with recurrent links to the main innate and adaptive immune populations. As shown by cell-type-pair interaction rankings derived from LIANA [46] (Fig R in S1 Appendix), both approaches identify cDC as one of the core communication hubs. However, LIANA assigns cDC a more dominant role because ligand–receptor evidence is concentrated in antigen-presentation programs and then aggregated to cell-type pairs, whereas scGSI yields a more uniform distribution that reflects global relationships in the integrated embedding space. Thus embedding similarity can capture biologically meaningful interaction tendencies at the cell-type level, while remaining distinct from direct molecular ligand–receptor inference.

In this dataset, the shared embedding remains interpretable at the level of cell-cell interaction structure while preserving both modality mixing and biological fidelity.

### scGSI enhances downstream cell-state resolution

We next examined downstream cell-state resolution on the TEA_PBMC dataset to test whether the integrated embeddings remain useful after alignment. Ground-truth cell-type labels for clustering accuracy evaluation on TEA_PBMC were the joint multi-omics annotations provided by the original TEA-seq study authors [47]. This dataset is informative because success requires more than modality mixing: the embedding must also preserve subtle subtype boundaries that support unsupervised clustering.

**Fig 4:**
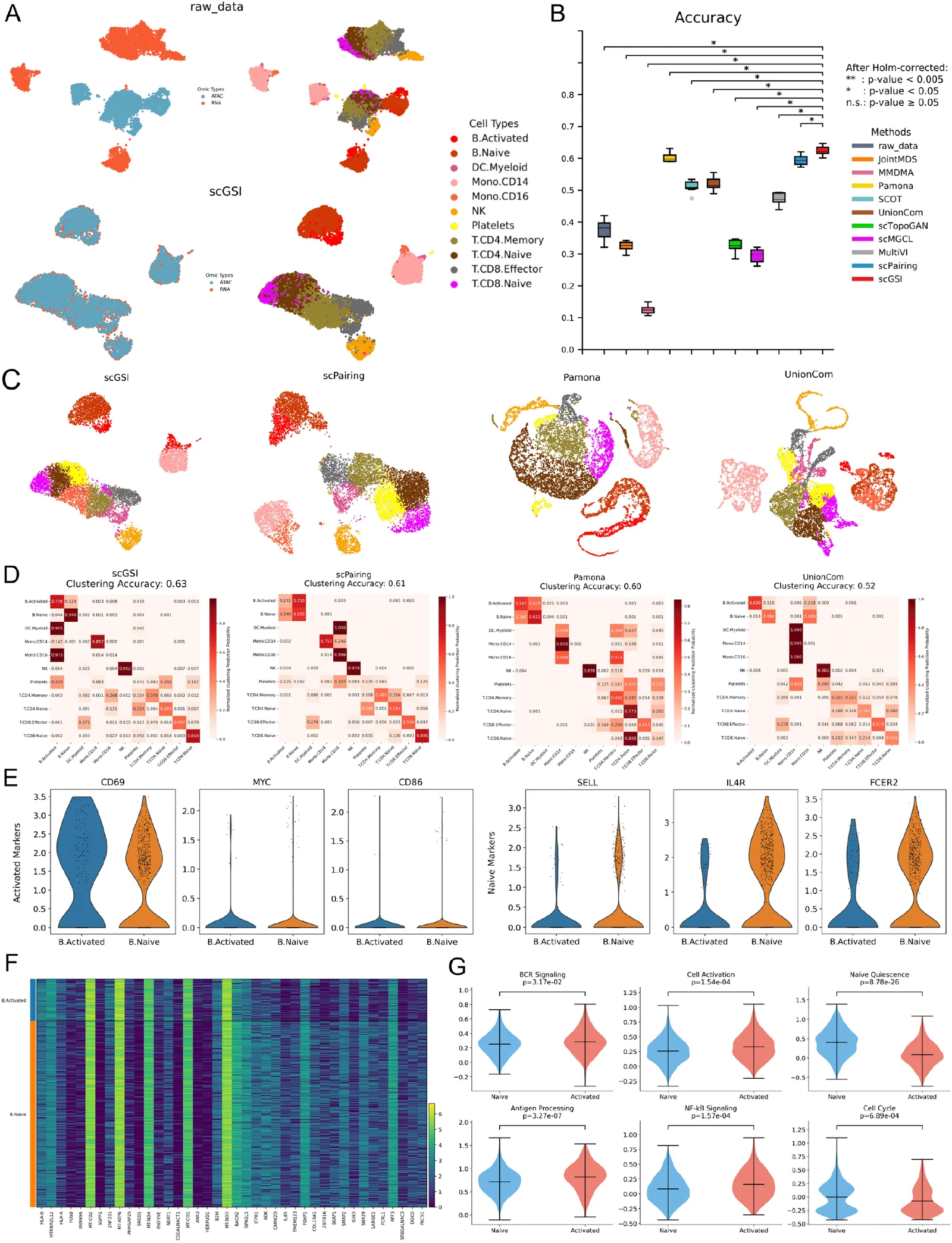
scGSI enhances downstream cell-state resolution on the TEA_PBMC dataset. Assessment of whether the integrated embeddings remain useful for downstream analysis by preserving fine-grained subtype boundaries after alignment. **(A)** UMAP visualization of the integrated embeddings. **(B)** Box plot of clustering accuracy, quantifying cell-state resolution after integration. Paired two-sided Wilcoxon signed-rank tests compare scGSI with each competing method; significance stars denote Holm-corrected *p*-values across all comparisons. **(C)** UMAP plots of K-means clustering results for scGSI, scPairing, Pamona, and UnionCom, illustrating differences in cluster compactness and subtype separation. **(D)** Heatmaps of clustering assignments for the same methods, showing misclassification structure across related immune lineages. **(E)** Violin plots of the top three marker genes for B.Naive and B.Activated, showing expression differences between the two groups. **(F)** Heatmap of the 40 most differentially expressed genes between the two groups, assessing whether the marker pattern is broadly supported. **(G)** GSEA of differentially expressed genes, testing co-enrichment in known B-cell functional pathways.

Fig 4A and Fig E in S1 Appendix show that the raw data contain substantial cell-type mixing. Among the competing methods, Pamona, SCOT, and JointMDS achieve only partial integration, whereas scPairing remains competitive but still fails to cleanly separate some biologically similar populations, such as B-cell states. By contrast, scGSI produces both strong modality alignment and sharper subtype boundaries, which suggests that the learned embedding preserves fine local structure rather than only improving global overlap.

We then quantified downstream utility through K-means clustering followed by optimal label matching. The box plot of clustering accuracy (Fig 4B) shows that scGSI achieves the highest scores. To assess statistical reliability, we performed paired two-sided Wilcoxon signed-rank tests on scGSI and each competing method. Every comparison yielded a nominal *p* < 0.05, and all differences remained significant after Holm correction across the full set of comparisons (Holm-adjusted *p* < 0.05). The clustering visualizations in Fig 4C and Fig F in S1 Appendix are consistent with this result: scGSI produces more compact and better-separated clusters, especially for biologically close subtypes such as B.Naive and B.Activated. The corresponding heatmap in Fig 4D and Fig G in S1 Appendix shows the strongest diagonal pattern among the leading methods, indicating less confusion across related immune lineages.

Some difficult cases remain, particularly among myeloid-derived populations such as DC.Myeloid and Mono.CD16, which likely reflect genuine developmental proximity rather than purely technical error. Even with these hard cases, scGSI achieves the strongest overall profile across metrics on this dataset (Fig P in S1 Appendix).

We conducted gene set enrichment analysis (GSEA) and differential expression analysis (DGE) on the highly similar B cell subsets of B.Naive and B.Activated to verify the accuracy of isolation at the transcriptome functional level [48,49]. The results showed that the B.Activated subpopulation successfully isolated by scGSI exhibited typical activation characteristics at multiple levels (Fig 4E): At the molecular marker level, the classical activation marker genes CD69, MYC and CD86 were significantly highly expressed in B.Activated [50,51], while the naive B cell marker genes SELL, IL4R and FCER2 were specifically enriched in B.Naive [52]. The whole transcriptome differential heatmap further confirmed this pattern (Fig 4F). The B.Activated subpopulation systematically upregulated antigen presentation related genes (HLA-A/B/C, B2M), AP.1 transcription factors (FOSB, JUN), and mitochondrial metabolic genes, reflecting enhanced antigen presentation function and metabolic reprogramming in the activated state [53,54]. Finally, GSEA analysis (Fig 4G) further verified this functional landscape and found that the B.Activated subgroup showed significant positive enrichment in core pathways such as Cell Activation, NF-κB signaling, and Antigen Processing. B.Naive is enriched in the Naive Quiescence and Cell Cycle pathways [55,56].

These analyses indicate that scGSI retains fine-grained cellular heterogeneity and that the recovered B-cell states remain consistent with established activation programs at the levels of transcriptional regulation and immunological function transition [53,54].

### scGSI recovers biologically coherent developmental trajectories

Trajectory inference provides a more demanding biological test than local alignment because it asks whether the integrated embedding preserves developmental organization. We therefore evaluated scGSI on the AdBraCor and P0BraCor brain cortex datasets using PAGA [57] and trajectory consistency analyses.

On the adult cortex dataset AdBraCor, scGSI again produces a well-mixed yet biologically structured embedding (Figs H–J in S1 Appendix). The PAGA graph of the raw data forms a dense web of cell-state connections in which key transitions are difficult to resolve, whereas after integration with scGSI, Mis cells emerge as clear bottleneck nodes that radiate into three major branches: excitatory, inhibitory, and glial-vascular. This organization is consistent with known cortical lineage structure [58, 59]. The full cross-method PAGA comparison for AdBraCor is provided in Fig I in S1 Appendix.

**Fig 5:**
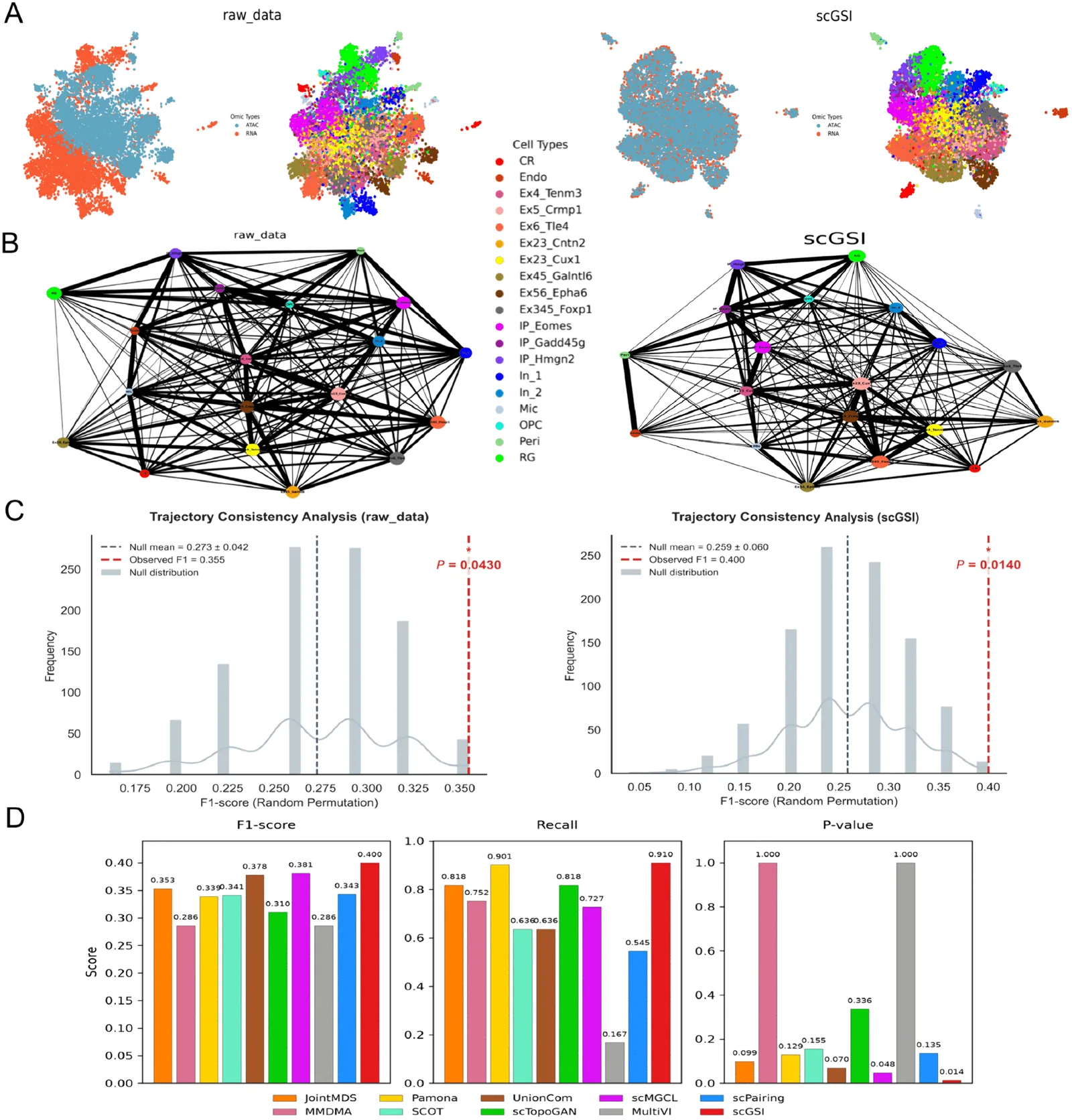
scGSI recovers biologically coherent developmental trajectories on the P0BraCor dataset. Assessment of whether the integrated embeddings preserve developmental organization beyond local geometric alignment. **(A)** UMAP visualization of the integrated embedding space. **(B)** PAGA trajectory inference graph, showing the developmental topology reconstructed from the scGSI embeddings. **(C)** Permutation test for trajectory consistency under a method-specific label-shuffle null. Nominal permutation *p*-values are shown for descriptive reference, and Holm- and Benjamini–Hochberg-adjusted *p*-values are provided in Table D in S1 Appendix. **(D)** Quantitative comparison of trajectory consistency across methods.

The neonatal cortex dataset P0BraCor makes this advantage even clearer. Compared with the raw-data topology and the broader cross-method comparison, scGSI reconstructs a more streamlined developmental topology with RG as the apical progenitor, consistent with post-natal cortical structure and pseudotime dynamics (Fig 5B and Figs K–L in S1 Appendix). This is especially relevant in post-natal brain analysis, where radial glial remnants and intermediate progenitors coordinate late neurogenesis and gliogenesis and where spurious lineage links can distort downstream regulatory interpretation.

Fig 5C–5D summarizes the trajectory consistency analysis. Among all evaluated methods, scGSI achieved the highest observed F1-score (0.400) and Recall (0.910), indicating the strongest preservation of developmental trajectory consistency. To complement this descriptive performance assessment, we used 1,000 random label permutations to evaluate whether the observed trajectory consistency score deviated from its own empirical null distribution. For scGSI (nominal *p*-value = 0.0140), the observed trajectory consistency score lies toward the upper tail of its empirical null distribution. Because multiple methods were evaluated under the same protocol, we applied multiple-testing correction across all evaluated methods (Table D in S1 Appendix). Adjusted *p*-values provide the statistical boundary for confirmatory inference, whereas the F1-score and Recall rankings remain the primary evidence supporting comparative trajectory preservation.

Across both adult and neonatal cortex data, scGSI preserves developmental organization in embedding geometry and in the ranking of trajectory consistency metrics.

### Ablation study

To identify which modules drive performance, we quantified the contribution of each component on the TEA_PBMC and P0BraCor datasets by systematically removing individual terms:

- **scGSI-w/o cfc**: removing the cross-fusion module.
- **scGSI-w/o clc**: removing the contrastive learning module.
- **scGSI-w/o pc**: removing the similarity projection.
- **scGSI-w/o gc**: removing the graph structure.

**Fig 6:**
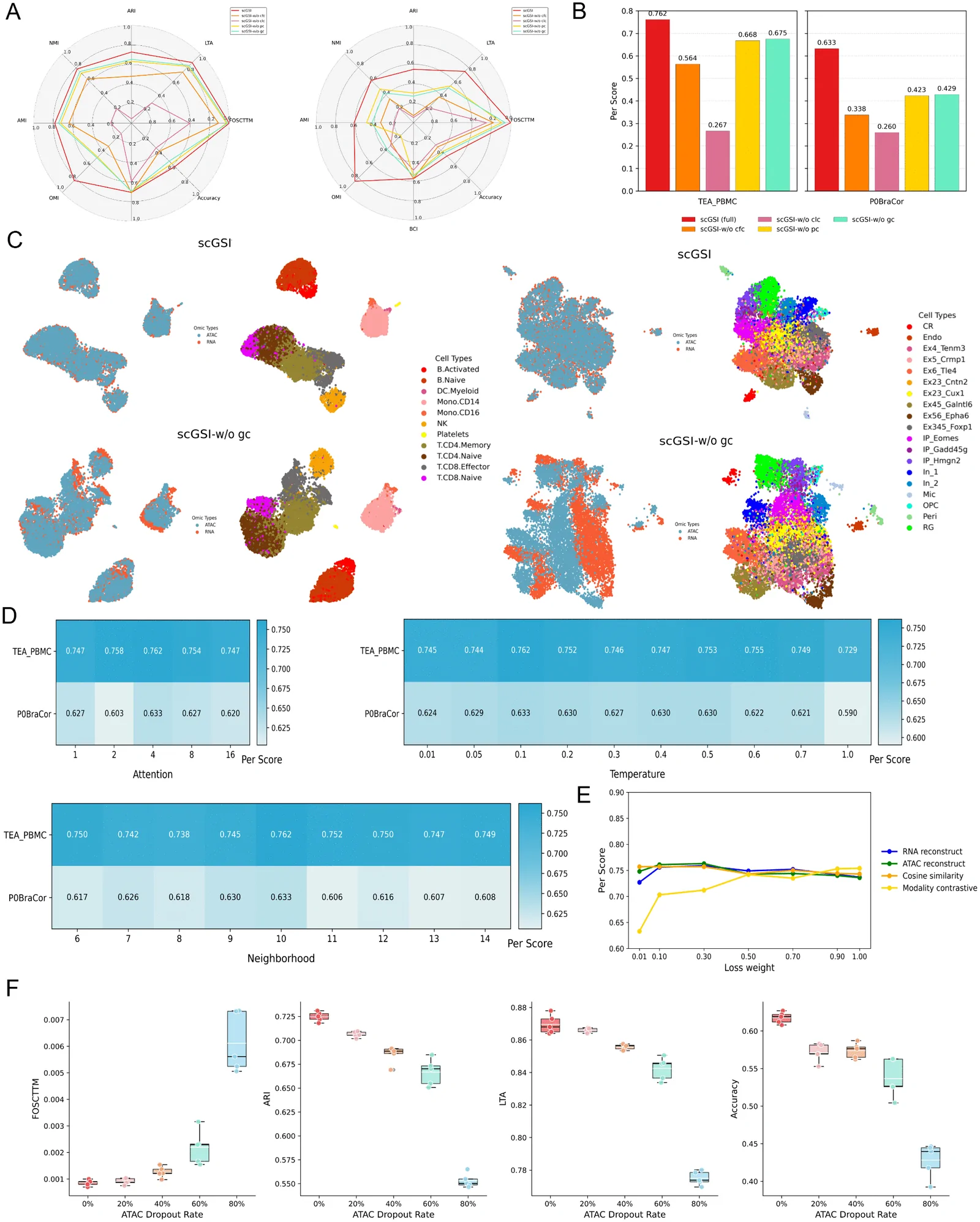
Ablation and robustness analyses explain why scGSI works and how stable it is. Contribution of each architectural component, sensitivity to key hyperparameters, and ATAC degradation robustness on the TEA_PBMC and P0BraCor datasets. **(A)** Radar plot comparing the performance of full scGSI and ablation variants across evaluation metrics. **(B)** Overall performance scores under different ablations, quantifying the contribution of graph structure, similarity projection, cross-fusion, and contrastive refinement. **(C)** Visual comparison between graph-based and non-graph variants, illustrating the role of structure-aware encoding in preserving integration quality and cluster boundaries. **(D)** Sensitivity to key hyperparameters, including the number of attention heads *Head*, the temperature parameter τ, and the number of nearest neighbors *k*. **(E)** Sensitivity to different loss weights. **(F)** Robustness under simulated ATAC feature dropout (0%–80%), where 0% denotes the undisturbed baseline.

Every ablation lowers performance across metrics (Fig 6A–6B), but the magnitude of the drop differs by module. Removing the cross-fusion module lowers the overall performance score by 0.198 on TEA_PBMC and 0.295 on P0BraCor, showing that explicit paired information exchange contributes substantially to the final embedding. Removing the contrastive learning module causes the largest decline, with overall performance drops of 0.498 and 0.373, respectively, indicating that contrastive refinement is the primary driver of paired-cell discrimination.

The structural modules provide an additional stabilizing effect. When graph structure is omitted, the GNN encoder reduces to a multi-layer perceptron and no longer models cell-cell relationships explicitly. This change leads to overall performance drops of 0.087 and 0.204, and the corresponding visualization shows weaker integration and blurred cluster boundaries (Fig 6C). Removing the similarity projection produces a decline of comparable magnitude, indicating that the graph encoder and the pre-alignment projection make complementary contributions. Taken together, the ablations suggest that contrastive refinement drives pair discrimination, whereas graph structure, similarity projection, and cross-fusion stabilize structure-aware alignment and downstream biological utility.

### scGSI is robust to hyperparameters and modality degradation

We next tested whether the gains of scGSI depend on narrow hyperparameter tuning. Specifically, we examined the sensitivity of the number of attention heads *Head*, the number of nearest neighbors *k*, and the temperature parameter τ on the TEA_PBMC and P0BraCor datasets.

As shown in Fig 6D and Fig M in S1 Appendix, performance is best at *Head* = 4 and declines only slightly when Head≤2 or ≥8, indicating good robustness. scGSI also performs best at τ=0.1 on both datasets. When τ is too small (τ≤0.05), the similarity distribution becomes overly sharp and alignment becomes unstable; when τ is too large (τ≥0.6), the distribution becomes too smooth and alignment quality declines. For the neighborhood parameter, performance remains stable across k∈[8,11], with purity fluctuations typically within ±0.04. Overall, the default configuration (*Head* = 4, τ=0.1, *k* = 10) remains near-optimal across a broad range of perturbations.

We also conducted a univariate loss-weight analysis on the TEA_PBMC dataset by varying one weight at a time while fixing the remaining three (Fig 6E and Fig N in S1 Appendix). The two reconstruction losses and the cosine-similarity loss are relatively robust, whereas the model is more sensitive to the modality-contrastive loss. Low weights on the modality-contrastive term lead to a marked performance decline, further emphasizing its role in distinguishing positive and negative sample pairs.

To evaluate the stability of scGSI under challenging data conditions, we simulated progressive degradation of ATAC modality quality on the TEA_PBMC dataset while keeping the RNA modality intact. Specifically, we randomly masked ATAC features at dropout rates of 0%, 20%, 40%, 60%, and 80% (0% denotes the undisturbed baseline) and repeated each rate with independent random masks. Fig 6F summarizes the resulting distributions as boxplots for FOSCTTM, ARI, LAT, and Accuracy. Under mild to moderate dropout (0%–60%), LAT remains largely stable and ARI declines only modestly, whereas Accuracy shows a clearer relative drop and FOSCTTM increases from a near-zero baseline. At 80% dropout, all four metrics degrade more markedly, yet scGSI still recovers usable cross-modal alignment rather than collapsing outright. These analyses indicate that scGSI does not rely on fragile parameter tuning and that its advantage remains visible under substantial ATAC quality loss.

## Discussion

We present scGSI, a graph-guided self-supervised fusion framework for paired single-cell multi-omics integration. The framework reconciles modality-specific topological structure by integrating pre-alignment proximity projection, cross-modal fusion coordination, and contrastive refinement. The central message of this work is that paired multi-omics integration should not be viewed as a pure alignment problem. Instead, a useful model must align corresponding cells while preserving the neighborhood structure and biological variation that make the embeddings informative for downstream analysis.

Although the current study primarily focuses on RNA-ATAC paired data where Signac enables a shared gene-level feature space, scGSI can be readily extended to other paired modalities such as CITE-seq (RNA + protein). In such cases, the two modalities possess completely non-overlapping feature spaces. The modality-specific projection heads in the pre-alignment module learn independent transformations for each modality, mapping them into a common latent space. This design, combined with the subsequent cross-fusion and contrastive learning, allows scGSI to handle heterogeneous feature spaces without requiring explicit feature name alignment.

Evaluations on five paired datasets against nine methods show that scGSI consistently improves this balance: it delivers strong paired cell-state matching, favorable modality mixing without severe biological erosion, and more useful embeddings for cell-type discrimination and developmental trajectory recovery. Beyond the performance gain itself, scGSI points to a practical design principle for paired data: preserve structure before aggressive alignment, use pairing to guide cross-modal complementarity, and constrain optimization so that alignment does not erase biology.

This design is particularly relevant for datasets in which subtle cell-state differences and developmental continuity must both be retained. On immune datasets, scGSI improves cross-modal matching while preserving subtype boundaries that remain difficult for many baseline methods. On brain cortex datasets, the integrated embeddings support more coherent lineage topology and more reliable trajectory recovery, so the improvement is not limited to cleaner visualization. Regarding computational efficiency, scGSI requires moderate runtime compared with lightweight baselines such as JointMDS and scPairing, but remains comparable to other graph-based and optimal-transport methods while achieving the lowest peak memory usage. This additional computation is accompanied by improved alignment accuracy and biological conservation, as demonstrated by the benchmark evaluations.

However, scGSI’s reliance on paired data limits its generalization to unpaired multi-omics integration, particularly in eliminating modality-specific batch effects. Moreover, BCI quantifies label-based conservation of cell-type organization in the integrated embedding rather than explicitly testing whether modality-specific local neighborhoods from the original RNA or ATAC spaces are preserved after fusion; neighborhood-overlap or trustworthiness-style metrics remain an important direction for future evaluation. In addition, the current study focuses mainly on RNA-centered paired settings, and biological interpretability is established primarily through downstream analyses rather than direct inspection of model internals. Certain closely related cell populations, such as continuous immune subtypes, also remain challenging because their underlying regulatory programs are intrinsically similar. Future work can therefore extend scGSI toward unpaired or batch-aware settings, incorporate spatiotemporal priors to refine developmental trajectory inference, and further characterize the biological meaning of learned cross-modal attention and graph structure.

Taken together, these design choices make scGSI a practical framework for paired single-cell multi-omics integration. Its cell-level analytical resolution may support applications ranging from heterogeneity dissection to therapeutic target discovery.

## Supporting information

S1 Appendix Supplementary methods and additional results for scGSI. **Table A. Dataset characteristics for the five paired single-cell multi-omics datasets used in this study.** Summary of species, tissue source, protocol, cell counts, cell types, and feature dimensions. **Table B. Default parameter settings used for scGSI across datasets.** Training configuration used for the main experiments and robustness analyses. **Table C. Source code repositories and software versions for all benchmark methods.** Repository URLs, version tags or commit hashes, and references for each method compared in this study. **Table D. Permutation test results for trajectory consistency across all benchmark methods.** Holm and Benjamini–Hochberg adjusted *p*-values for trajectory F1-score permutation tests on the P0BraCor dataset. **Fig A. Full UMAP comparison across methods for BMMC dataset.** UMAP visualizations colored by modality and cell type for all integration methods on BMMC dataset. **Fig B. Full ATAC-to-RNA label transfer confusion matrices for BMMC dataset.** Comparison of label transfer accuracy across all methods on BMMC dataset. **Fig C. PAGA inference visualizations for all methods on the BMMC dataset.** Comparison of lineage topology recovered by different integration methods on the BMMC dataset. **Fig D. UMAP comparison across methods for CITE_PBMC dataset.** UMAP visualizations colored by modality and cell type for all integration methods on CITE_PBMC dataset. **Fig E. UMAP comparison across methods for TEA_PBMC dataset.** UMAP visualizations colored by modality and cell type for all integration methods on TEA_PBMC dataset. **Fig F. Clustering UMAP visualization for all methods on TEA_PBMC dataset.** Comparison of K-means clustering results across all integration methods. **Fig G. Heatmaps of K-means clustering results for all methods on the TEA_PBMC dataset.** Heatmap comparison of cluster assignments across methods. **Fig H. UMAP comparison across methods for the AdBraCor dataset.** UMAP visualizations colored by modality and cell type for all integration methods on the AdBraCor dataset. **Fig I. PAGA inference visualizations for all methods on the AdBraCor dataset.** Comparison of lineage topology recovered by different integration methods on the AdBraCor dataset. **Fig J. Integration performance of scGSI on the AdBraCor dataset.** UMAP, PAGA, and quantitative summary panels for scGSI on adult cortex data. **Fig K. UMAP comparison across methods for the P0BraCor dataset.** UMAP visualizations colored by modality and cell type for all integration methods on the P0BraCor dataset. **Fig L. PAGA inference visualizations for all methods on the P0BraCor dataset.** Comparison of developmental topology recovered by different integration methods on the P0BraCor dataset. **Fig M. Hyperparameter sensitivity analysis.** Full sensitivity analysis for attention heads, nearest neighbors, and temperature parameter. **Fig N. Loss weight sensitivity analysis.** Univariate loss-weight analysis on TEA_PBMC dataset. **Fig O. Encoder selection analysis.** Additional analysis comparing different graph encoder choices for each modality. **Fig P. Dataset-specific radar charts and overall performance box plots across all datasets.** Comprehensive benchmark summary comparing scGSI to all baseline methods across the five benchmark datasets. Performance distributions are shown as box plots across repeated runs. **Fig Q. Differential enrichment scores of B-cell subsets across key biological pathways.** Bar-chart summary of pathway enrichment differences between B.Activated and B.Naive cells. **Fig R. Bar chart of the top 20 cell-type-pair interaction propensities inferred with LIANA.** Each bar represents a cell-type pair ranked by aggregated interaction propensity derived from LIANA ligand–receptor analysis. **Fig S. Runtime and peak memory usage across datasets.** Median runtime (log_10_ scale) and peak memory consumption of all benchmarked methods across the five datasets, with per-dataset values overlaid; scGSI is highlighted in red.
(PDF)
