# Supplementary material for: scGSI: Graph-guided self-supervised integration of paired single-cell multi-omics
Source: PLoS Comput Biol. 2026 Sep 8;22(9):e1014773. doi: 10.1371/journal.pcbi.1014773 (PMC13577519; doi:10.1371/journal.pcbi.1014773)
Supplement: Supplementary file 1 [file pcbi.1014773.s001.pdf]

Supplementary Materials for "scGSI: Graph-guided self-supervised integration of paired single-cell multi-omics"

Xiang Chen[1,2,*], Zihan Yang[1], Xiaoyu Liu[1], Zhiyi Xie[1], and Wenlu Guo[1]

[1]School of Computer Science and Engineering, Hunan University of Science and Technology, Xiangtan, Hunan, China.
[2]Hunan Provincial Key Laboratory of Intelligent Computing and Language Information Processing, Hunan University of Science and Technology, Xiangtan, Hunan, China.
[*]Corresponding author: chenxofhit@gmail.com

1 Supplementary Notes

1.1 Transformation of gene activity score matrices

This section details the biologically informed feature-bridging step used by scGSI before paired alignment. Because scRNA-seq and scATAC-seq are measured in fundamentally different feature spaces, direct comparison between the two modalities is unstable unless a shared representation is first established. We therefore transform the scATAC-seq peak matrix into a gene activity score matrix that can be aligned with scRNA-seq while retaining accessibility-derived regulatory information.

Existing approaches mainly differ in how they model peak-gene associations and how they weight the contribution of accessible regions to gene-level activity:

- **ArchR[1]**: Uses latent semantic analysis (LSA) to reduce the peak matrix dimensionality, computing peak-gene scores based on TF-IDF weights and gene annotations. The weighting mechanism integrates peak intensity with genomic position for high-resolution activity estimation, with computational complexity of $O(cp)$, where c is the number of cells and p is the number of peaks.
- **Cicero[2]**: Constructs a loop graph model via a hidden Markov model (HMM) to infer enhancer-gene linkages, with activity scores derived from weighted peak aggregation ($A_g = \sum_p W_{pg} s_p$, where W_{pg} stems from linkage probabilities). This approach captures long-range regulation but relies on Hi-C priors, making it suitable for reference-guided scenarios.
- **scATAC-pro[3]**: For gene activity estimation, scATAC-pro provides a Cicero-based module (runCicero) that constructs a loop graph model via a hidden Markov model (HMM) to infer enhancer-gene linkages and compute gene activity scores ($A_g = \sum_p W_{pg} s_p$, where W_{pg} stems from linkage probabilities). It also offers a label transfer module that constructs a gene activity matrix from scATAC-seq data for cross-modal annotation using the Seurat framework.
- **Signac[4]**: Computes gene activity scores by counting ATAC fragments that overlap each gene body together with a proximal upstream promoter window (commonly on the order of 2 kb), producing a cell-by-gene accessibility matrix that shares a gene-level feature space with scRNA-seq. This fragment-counting formulation is computationally efficient, integrates readily with the Seurat ecosystem, and is distinct from ChromVAR motif-deviation scoring.

We adopted Signac because it offers a favorable balance among computational efficiency ($O(pN)$, where N is the number of cells), ease of standardization, and compatibility with RNA-centered downstream analysis. Compared with alternative tools such as ArchR, Signac provides a streamlined gene-activity implementation that avoids additional motif- or loop-model optimization while remaining compatible with standard single-cell analysis workflows. This choice is consistent with the overall design principle of scGSI: introduce a stable and biologically meaningful bridge between modalities before imposing aggressive alignment. After transformation, we apply log-normalization to the resulting matrix so that the two modalities enter graph construction on more comparable scales.

1.2 Data preprocessing

To preserve modality-specific structure before integration, we preprocess scRNA-seq and scATAC-seq independently rather than forcing them into an identical normalization pipeline. This strategy reflects the main logic of scGSI: each modality should first be denoised and summarized according to its own statistical properties, and only then should paired alignment be performed. The preprocessing pipelines, implemented in Scanpy [5] and scikit-learn [6], were executed through the custom functions *preprocess_rna* and *preprocess_atac*. They produce low-dimensional modality-specific representations that serve as the input to graph construction and downstream encoding.

For scRNA-seq data, we use a dedicated preprocessing procedure to extract high signal-to-noise gene expression features while preserving transcriptional continuity. First, Scanpy's *sc.pp.highly_variable_genes* (Seurat V3 algorithm) selects the top 2000 highly variable genes based on expression variability. Then, *sc.pp.normalize_total* performs total-count normalization to 10,000 counts per cell, followed by *sc.pp.log1p* log-transformation ($\log(x+1)$) to stabilize variance and compress the dynamic range of expression. Finally, we apply zero-centered scaling to each gene (mean of zero, standard deviation of one) and perform PCA dimensionality reduction (ARPACK solver) to extract the top 50 principal components as low-dimensional cell representations.

For scATAC-seq data, the *preprocess_atac* function transforms chromatin accessibility measurements into gene activity-oriented representations that can be compared with RNA while remaining robust to sparsity. We first apply TF-IDF transformation to weight the peak count matrix by computing term frequency (TF = counts / total peaks per cell) and inverse document frequency (IDF = log(total cells / cells containing the peak)), yielding a weighted matrix (TF $\times$ IDF) that amplifies signals from cell-specific open regions while suppressing noise. This is followed by L1 normalization and log-transformation ($\mathrm{log1p}(\mathrm{tfidf} \times 10^4)$) of the TF-IDF matrix. For dimensionality reduction, we use randomized singular value decomposition (SVD) via scikit-learn to extract the top 50 latent semantic indexing (LSI) components, followed by zero-centering and standard deviation scaling. The resulting LSI coordinates are used as the low-dimensional embeddings for scATAC-seq data.

1.3 Graph construction for cell matching

This section describes the graph construction module that supports structure-aware encoding in scGSI. The goal of this module is to capture local cellular topology within each modality before any cross-modal fusion is introduced. To do so, we construct directed or undirected graphs using the k-nearest neighbors (KNN) algorithm, with support for either connectivity mode or distance mode and compatibility with multiple distance metrics, including Minkowski, Jaccard, and correlation. Let $X \in \mathbb{R}^{N\times D}$ denote a single-modality input matrix. Selecting the optimal metric and neighbor number k, we first compute the pairwise distance matrix $D \in \mathbb{R}^{N\times N}$, where D_{ij} is defined as:

$$D_{ij} = \begin{cases} \|x_i - x_j\|_p & \text{if metric = “minkowski”}, \\ 1 - \frac{|S_i \cap S_j|}{|S_i \cup S_j|} & \text{if metric = “jaccard”}, \\ 1 - \mathrm{corr}(x_i, x_j) & \text{if metric = “correlation”} \end{cases} \tag{1}$$

where x_i, x_j are feature vectors of cells i and j, $\|\cdot\|_p$ is the L_p norm, S_i denotes the support set (i.e., the set of indices of all detected/non-zero features) of cell i in the current modality, S_j is defined analogously for cell j, and corr is the correlation coefficient. Subsequently, for each cell i, we select the nearest $k+1$ neighbors (including itself) to construct the local neighborhood set $N_i = \{j | j \in \text{top-k}(d_i) \setminus \{i\}\}$. If graph mode is “connectivity”, we generate a binary adjacency matrix $A \in \mathbb{R}^{N\times N}$:

$$A_{ij} = \begin{cases} 1 & \text{if } j \in N_i, \\ 0 & \text{otherwise} \end{cases} \tag{2}$$

converted to edge indices $E = \{(i,j) | A_{ij} = 1\}$ and an unweighted graph $G = (V, E)$ ($V = \{1, ..., N\}, E = E \cup E^T$ for undirected). If graph mode is “distance”, when constructing E we skip the data point itself (first neighbor), generating a weighted adjacency matrix $W \in \mathbb{R}^{N\times N}$:

$$W_{ij} = \begin{cases} d_{ij} & \text{if } j \in N_i, \\ \infty \text{ or } 0 & \text{otherwise} \end{cases} \tag{3}$$

outputting weighted edges $G = (E, W_E)$ (directed), where $W_E = \{d_{ij} | (i,j) \in E\}$.

In practice, this graph serves as the structural scaffold used by the modality-specific GNN encoders. By encoding cells together with their local neighborhoods, scGSI preserves within-modality geometry before attempting paired alignment across modalities.

1.4 Integration performance metrics

Because the goal of scGSI is broader than simple modality mixing, we evaluate performance from several complementary perspectives. The metrics below are organized to reflect the same logic used in the main text: paired alignment accuracy, cell-state discrimination, modality mixing, and biological conservation. Together, they let us judge not only whether the two modalities are mixed, but also whether the resulting embeddings remain biologically meaningful and useful for downstream analysis.

1.4.1 FOSCTTM

The Fraction of Samples Closer Than True Matches (FOSCTTM) assesses the alignment quality of single-cell multimodal embeddings. It quantifies the relative position of each cell's "true match" within the ranked neighborhood, measuring the local consistency of the embedding space (lower values are better, with 0 indicating perfect alignment). This metric is computed bidirectionally (RNA → ATAC and ATAC → RNA) and averaged to ensure symmetry.

$$\text{FOSCTTM} = \frac{1}{2}\left[\frac{1}{N}\sum_{i=1}^{N}\frac{r_i^{\text{RNA}\to\text{ATAC}}}{N-1} + \frac{1}{N}\sum_{i=1}^{N}\frac{r_i^{\text{ATAC}\to\text{RNA}}}{N-1}\right] \tag{4}$$

where $r_i^{\text{RNA}\to\text{ATAC}} = r_i$ denotes the rank position (starting from 0) of the true match (itself) for cell i in the Euclidean distance sorting to all ATAC cells, and analogously for $r_i^{\text{ATAC}\to\text{RNA}}$; N is the number of cells.

1.4.2 Label Transfer Accuracy

Label Transfer Accuracy (LAT) evaluates the alignment quality of multimodal embeddings by using a k-nearest neighbors (KNN) classifier to propagate labels from one modality to another within the shared embedding space. Values range from 0 to 1, with higher scores indicating more precise cross-modal correspondence of cell states.

$$\text{LAT} = \frac{1}{N_1}\sum_{i=1}^{N_1}\mathbb{I}\left(\hat{y}_i^{(1)} = y_i^{(1)}\right) \tag{5}$$

where $\hat{y}_i^{(1)}$ denotes the predicted label for $X^{(1)}$ obtained via a KNN classifier trained on $(X^{(2)}, y^{(2)})$, $y_i^{(1)}$ is the ground-truth label for $X^{(1)}$, and N_1 is the number of samples in the target modality.

1.4.3 Adjusted Rand Index

Adjusted Rand Index (ARI) quantifies the agreement between predicted clustering results and ground-truth labels, measuring the similarity between two clusterings while accounting for chance agreements. It ranges from -1 to 1, where 1 indicates perfect agreement, 0 represents random labeling, and negative values denote anti-correlation. The formula is given by:

$$\text{ARI} = \frac{\text{RI}(U,V) - \mathbb{E}[\text{RI}(U,V)]}{\max(\text{RI}(U,V)) - \mathbb{E}[\text{RI}(U,V)]} \tag{6}$$

where $\text{RI}(U,V)$ is the Rand Index, quantifying the pairwise agreement of sample pairs between clusterings U and V; $\mathbb{E}[\text{RI}(U,V)]$ is the expected value of RI; and $\max(\text{RI}(U,V))$ is the maximum value of RI.

1.4.4 Normalized Mutual Information

Normalized Mutual Information (NMI) evaluates the consistency between predicted clustering results and ground-truth labels by measuring their shared information, with entropy-based normalization to mitigate biases from varying cluster counts. It ranges from 0 (no agreement) to 1 (perfect agreement).

$$\text{NMI} = \frac{2\cdot I(X,Y)}{H(X)+H(Y)} \tag{7}$$

where $I(X,Y)$ denotes the mutual information between the predicted labels X and the ground-truth labels Y, while $H(X)$ and $H(Y)$ represent the entropies of X and Y, respectively.

1.4.5 Adjusted Mutual Information

Adjusted Mutual Information (AMI) quantifies the agreement between predicted labels and ground-truth labels by adjusting mutual information for chance occurrences, mitigating biases from differences in the number of predicted clusters. It ranges from -1 (anti-correlation) to 1 (perfect agreement), with 0 indicating random alignment.

$$\text{AMI} = \frac{I(U;V) - \mathbb{E}[I(U;V)]}{\frac{H(U)+H(V)}{2} - \mathbb{E}[I(U;V)]} \tag{8}$$

where $I(U;V)$ denotes the mutual information between the predicted clustering U and the ground-truth clustering V, $H(U)$ and $H(V)$ represent their respective entropies, and $\mathbb{E}[I(U;V)]$ is the expected mutual information under random partitions.

1.4.6 Clustering Accuracy

Clustering Accuracy (Accuracy) evaluates the agreement between unsupervised clustering outcomes and ground-truth labels, computed via adjusted predicted labels (using Hungarian algorithm) to measure classification correctness under optimal matching. It ranges from 0 to 1, with 1 indicating perfect alignment.

Let $y \in \{1, \ldots, K\}^N$ denote the ground-truth labels, $\hat{y}$ the adjusted predicted labels (via Hungarian algorithm mapping from K-means clustering), N the number of samples, and K the number of clusters. Accuracy is defined as:

$$\text{Accuracy} = \frac{1}{N}\sum_{i=1}^{N} \mathbb{C}(\hat{y}_i = y_i) \tag{9}$$

where $\mathbb{C}(\cdot)$ is the indicator function (1 if matched, 0 otherwise). This computation ensures maximal consistency following label permutation.

1.4.7 Omics Mixing Index

- **Neighborhood Overlap Score (NOS)** quantifies the local alignment quality between two embedding spaces by measuring the proportion of "true matches" (cross-modal correspondences for the same cell) within k-nearest neighbor (KNN) neighborhoods. It is the average of the directional overlap ratios between the two modalities, ranging from 0 (no overlap) to 1 (perfect local alignment).

$$\text{NOS} = \frac{1}{2N}\sum_{i=1}^{N} \left(\mathbb{Q}\left(i + N \in \text{NN}_k(i;X)\right) + \mathbb{Q}\left(i \in \text{NN}_k(i+N;X)\right)\right) \tag{10}$$

  where $X = [Z_{\text{RNA}}; Z_{\text{ATAC}}] \in \mathbb{R}^{2N\times D}$, with N the number of cells and D the embedding dimension; $\text{NN}_k(i;X)$ denotes the set of k nearest neighbor indices for cell i in X (Euclidean distance, excluding itself); and $\mathbb{Q}(\cdot)$ is the indicator function.
- **Graph Connectivity (GC)** assesses the internal cohesion of cell type subgroups within a graph constructed from the k nearest neighbors of each cell in the embedding space. It ranges from 0 to 1, where 1 indicates that all cells within each type form a single fully connected component.

$$\text{GC} = \frac{1}{K}\sum_{k=1}^{K} \frac{\max(|C_k|)}{|Y_k|} \tag{11}$$

  where K is the number of unique cell types in y, Y_k is the set of cells of the k-th type, and C_k denotes the connected components in the subgraph induced by Y_k (computed via strong connectivity from the Scanpy adjacency matrix).
- **Seurat Alignment Score (SAS)** quantifies the quality of modality integration in embeddings by evaluating the mixing of modality labels within local neighborhoods through repeated subsampling to balance modality sizes. It ranges from 0 to 1, where 1 denotes ideal mixing (no modality bias in neighbors) and 0 indicates complete segregation.

$$\text{SAS} = \frac{1}{R}\sum_{r=1}^{R} \min\left((1 - \frac{\bar{s}_r}{k}) \cdot \frac{B}{B-1}, 1\right) \tag{12}$$

Here, $R = n_{\text{repeats}}$ denotes the number of subsampling iterations, $B = |\text{unique}(y)|$ the number of modalities, with each subsample drawing $\min(|y|)$ cells per modality (yielding a total of $N' = B \cdot \min(|y_b|)$ subsampled cells), and $k = \max(\lceil N' \cdot \text{neighbor\_frac} \rceil, 1)$ the neighborhood size. For the r-th subsample, $\bar{s}_r$ represents the average count of same-modality neighbors (excluding self) across all subsampled cells.

Omics Mixing Index (OMI) measures the uniformity of fusion between modalities by averaging multiple complementary evaluation metrics. It ranges from 0 to 1, with higher values indicating enhanced integration and consistency across modalities.

$$\text{OMI} = \frac{\text{NOS} + \text{GC} + \text{SAS}}{3} \tag{13}$$

1.4.8 Biological Conservation Index

- **Average Silhouette Width (ASW)** quantifies the separation and cohesion of cell type clusters within coordinate embeddings by averaging the silhouette coefficients across all cells, assessing how well each cell aligns with its assigned type relative to adjacent clusters. It ranges from 0 to 1, where 1 indicates fully distinct and compact clusters.

$$\text{ASW} = \frac{1}{N} \sum_{i=1}^{N} s(i) \tag{14}$$

$$s(i) = \frac{b(i) - a(i)}{\max\{a(i), b(i)\}} \tag{15}$$

  where $s(i)$ denotes the silhouette width for cell i, $a(i)$ is the average Euclidean distance from i to other cells within its cluster A (based on coordinates x), and $b(i)$ is the minimum average distance from i to cells in any other cluster $B \neq A$.
- **Purity Score (PS)** evaluates the local clustering homogeneity within embeddings by averaging the proportion of the k nearest neighbors that share the same cell type label across all cells. It ranges from 0 to 1, where 1 indicates complete purity (all neighbors match the cell's type) and 0 indicates complete mixing.

$$\text{PS} = \frac{1}{N} \sum_{i=1}^{N} \frac{1}{k} \sum_{j \in \mathcal{N}_i} \mathbf{1}(\ell_j = \ell_i) \tag{16}$$

  where N is the number of cells; $\mathcal{N}_i$ denotes the indices of the k nearest neighbors of cell i (excluding itself, based on Euclidean distance); ℓ_i is the label of cell i; and $\mathbf{1}(\cdot)$ is the indicator function.
- **Mean Average Precision (MAP)** evaluates clustering quality in an embedding by quantifying the retrieval accuracy of same-cell-type instances within ranked lists of the k nearest neighbors. It ranges from 0 to 1, where 1 indicates perfect retrieval (all neighbors match the cell's type, in order).

$$\text{MAP} = \frac{1}{N} \sum_{i=1}^{N} \text{AP}_i \tag{17}$$

  Here, $N = |y|$ is the number of cells, $k = \max(\lceil N \cdot \text{neighbor\_frac} \rceil, 1)$ defines the neighborhood size, and for each cell i, AP_i is the average precision score among its k nearest neighbors (excluding self) in the coordinate space x.

Biological Conservation Index (BCI) is the average of the above three sub-metrics. Importantly, ASW, PS, and MAP are computed in the *integrated* embedding using cell-type labels; they quantify label-based conservation of cell-type organization rather than the preservation of modality-specific kNN neighborhoods from the original RNA or ATAC feature spaces. Higher BCI scores therefore indicate better retention of cell-type structure after integration, but they should not be interpreted as a direct test of cross-space neighborhood fidelity.

$$\text{BCI} = \frac{\text{ASW} + \text{PS} + \text{MAP}}{3} \tag{18}$$

1.5 Baseline methods

To contextualize the position of scGSI within the current paired and general multi-omics integration landscape, we compared it with representative methods spanning manifold alignment, optimal transport, adversarial alignment, graph contrastive learning, and variational co-embedding. Rather than reproducing the full implementation details of each baseline, we summarize below the main modeling idea of each method and the aspect most relevant to our benchmark setting.

- **UnionCom[7]** is a topology-preserving manifold alignment method that matches datasets by comparing their intrinsic geometric structures without requiring explicit cell or feature correspondences. Its main strength lies in preserving global manifold organization, but because it is designed for correspondence-free alignment, it does not explicitly exploit the within-cell pairing information available in our setting.
- **scTopoGAN[8]** aligns modalities through adversarial learning while encouraging consistency of topological features derived from neighborhood graphs. It is well suited to nonlinear manifold matching, but its adversarial formulation targets global cross-domain alignment rather than explicit paired-cell discrimination.
- **JointMDS[9]** combines multidimensional scaling with Wasserstein-Procrustes alignment to jointly infer embeddings and correspondences across domains. It provides a principled geometry-based alignment baseline, but, like other general manifold methods, it relies mainly on cross-dataset distance structure rather than paired multimodal complementarity.
- **Pamona[10]** formulates integration as a partial manifold alignment problem using partial Gromov-Wasserstein optimal transport, allowing shared and dataset-specific structures to coexist. This flexibility is valuable when modalities only partially overlap, but the method is still driven primarily by transport-based geometry matching rather than by explicit paired feature interaction.
- **MMD-MA[11]** aligns datasets by minimizing maximum mean discrepancy while preserving modality-specific geometry through regularized low-dimensional projections. It is an important distribution-matching baseline, although its objective is more appropriate for global alignment than for fine-grained paired cell matching.
- **SCOT[12]** uses unbalanced optimal transport to align cells across modalities while tolerating differences in abundance and dataset composition. Its transport-based design is useful for correspondence inference under imbalance, but it does not directly leverage known pairing information at the cell level.
- **scMGCL[13]** is a multi-view graph contrastive learning framework that integrates omics modalities through graph-based encoders and cross-view contrastive objectives. Among the graph-based baselines, it is the one most closely related to scGSI in spirit, but it emphasizes cross-view graph augmentation and contrastive agreement more than explicit paired cross-modal feature fusion.
- **MultiVI[14]** is a variational autoencoder-based framework that jointly models scRNA-seq and scATAC-seq data in a shared latent space using a product-of-experts inference network. It provides a probabilistic co-embedding baseline with explicit modeling of modality-specific noise, though its focus on generative reconstruction differs from the graph-structured alignment strategy of scGSI.
- **scPairing[15]** is a CLIP-inspired contrastive co-embedding framework that maps modality-specific representations into a shared spherical latent space and explicitly aligns paired cells. It is one of the most competitive paired-data baselines in our benchmark because it directly exploits cell-level correspondence, but its shared latent formulation is more focused on pair alignment than on preserving modality-specific neighborhood structure through graph-aware encoding.

2 Supplementary tables and figures

Table A. Dataset characteristics for the five paired single-cell multi-omics datasets used in this study. For each dataset, we report the species, tissue source, sequencing protocol, number of cells, annotated cell types, and feature dimensions to summarize the scale and diversity of the benchmark collection.

Dataset	Species	Tissue source	Protocol	Cells	Cell types	Genes	Peaks
BMMC	Homo sapiens	Bone marrow mononuclear cells	10x Multiome	6,224	21	13,381	116,490
CITE_PBMC	Homo sapiens	Peripheral blood mononuclear cells	CITE-seq	9,631	19	20,955	107,194
TEA_PBMC	Homo sapiens	Peripheral blood mononuclear cells	TEA-seq	5,041	11	9,955	14,732
AdBraCor	Mus musculus	Brain cortex	SNARE-seq	10,309	23	33,160	244,544
P0BraCor	Mus musculus	Brain cortex	SNARE-seq	5,081	19	18,322	229,439

Table B. Default parameter settings used for scGSI across datasets. The search ranges for hyperparameters are set as: Epoch ∈ [100, 200, 300, 400, 500], k ∈ [6, 7, 8, 9, 10, 11, 12, 13, 14], Lr ∈ [0.001, 0.003, 0.005, 0.01], Dropout ∈ [0.01, 0.05, 0.1], τ ∈ [0.01, 0.05, 0.1, 0.2, 0.3, 0.4, 0.5, 0.6, 0.7, 1.0], Head ∈ [1, 2, 4, 8, 16]. Even when a uniform Epoch upper limit is applied across datasets, differences in validation-loss convergence can lead to slight variation in the final number of epochs, while the remaining parameters remain consistent across all datasets. These values define the training configuration used in the main experiments and provide the reference setup for the robustness analyses reported in the supplementary figures.

Dataset	Latent dim	Epoch	k	Lr	Dropout	τ	$Head$
BMMC	32	450	10	0.003	0.01	0.1	4
CITE_PBMC	32	150	10	0.003	0.01	0.1	4
TEA_PBMC	32	300	10	0.003	0.01	0.1	4
AdBraCor	32	150	10	0.003	0.01	0.1	4
P0BraCor	32	150	10	0.003	0.01	0.1	4

Table C. Source code repositories and software versions for all benchmark methods used in this study. For each method, we report the repository URL and the latest release tag (or commit hash when no release is available), retrieved on 23 July 2026. Baseline methods were run with the author-recommended default parameter settings from the original publications and code repositories; no additional grid search was performed for the baselines. Hyperparameter search ranges and selected defaults for scGSI are reported in Supplementary Table B.

Method	URL	Version / commit	Ref.
JointMDS	`https://github.com/BorgwardtLab/JointMDS`	419c856	[9]
MMDMA	`https://bitbucket.org/noblelab/2019_mmd_wabi/src/master/`	8921b4e	[11]
Pamona	`https://github.com/caokai1073/Pamona`	v0.1.0	[10]
SCOT	`https://github.com/rsinghlab/SCOT`	SCOTv1.0	[12]
UnionCom	`https://github.com/caokai1073/UnionCom`	v0.4.0	[7]
scTopoGAN	`https://github.com/AkashCiel/scTopoGAN`	fb99533	[8]
scMGCL	`https://github.com/zlCreator/scMGCL`	374b5fd	[13]
MultiVI	`https://github.com/scverse/scvi-tools`	scvi-tools 1.5.0.post1	[14]
scPairing	`https://github.com/Ding-Group/scPairing`	65b73be	[15]

Table D. Nominal and multiplicity-adjusted permutation p-values for trajectory consistency on P0BraCor. For each of the 10 integration methods shown in Fig. 5D, the nominal p-value is obtained from 1,000 random label permutations of the trajectory F1-score. The unintegrated raw-data result shown in Fig. 5C is retained as a descriptive reference and is not included in this 10-method testing family. Holm-adjusted p-values control the family-wise error rate, and Benjamini–Hochberg (BH) adjusted p-values control the false discovery rate. After adjustment, no method remains below $p = 0.05$; the observed F1-score and Recall rankings are therefore interpreted descriptively as the primary cross-method comparison.

Method	Nominal p	Holm p	BH p
scGSI	0.0140	0.1400	0.1400
scMGCL	0.0480	0.4320	0.2214
UnionCom	0.0700	0.5600	0.2214
JointMDS	0.0990	0.6930	0.2214
Pamona	0.1290	0.7740	0.2214
scPairing	0.1350	0.7740	0.2214
SCOT	0.1550	0.7740	0.2214
scTopoGAN	0.3360	1.0000	0.4200
MultiVI	1.0000	1.0000	1.0000
MMDMA	1.0000	1.0000	1.0000

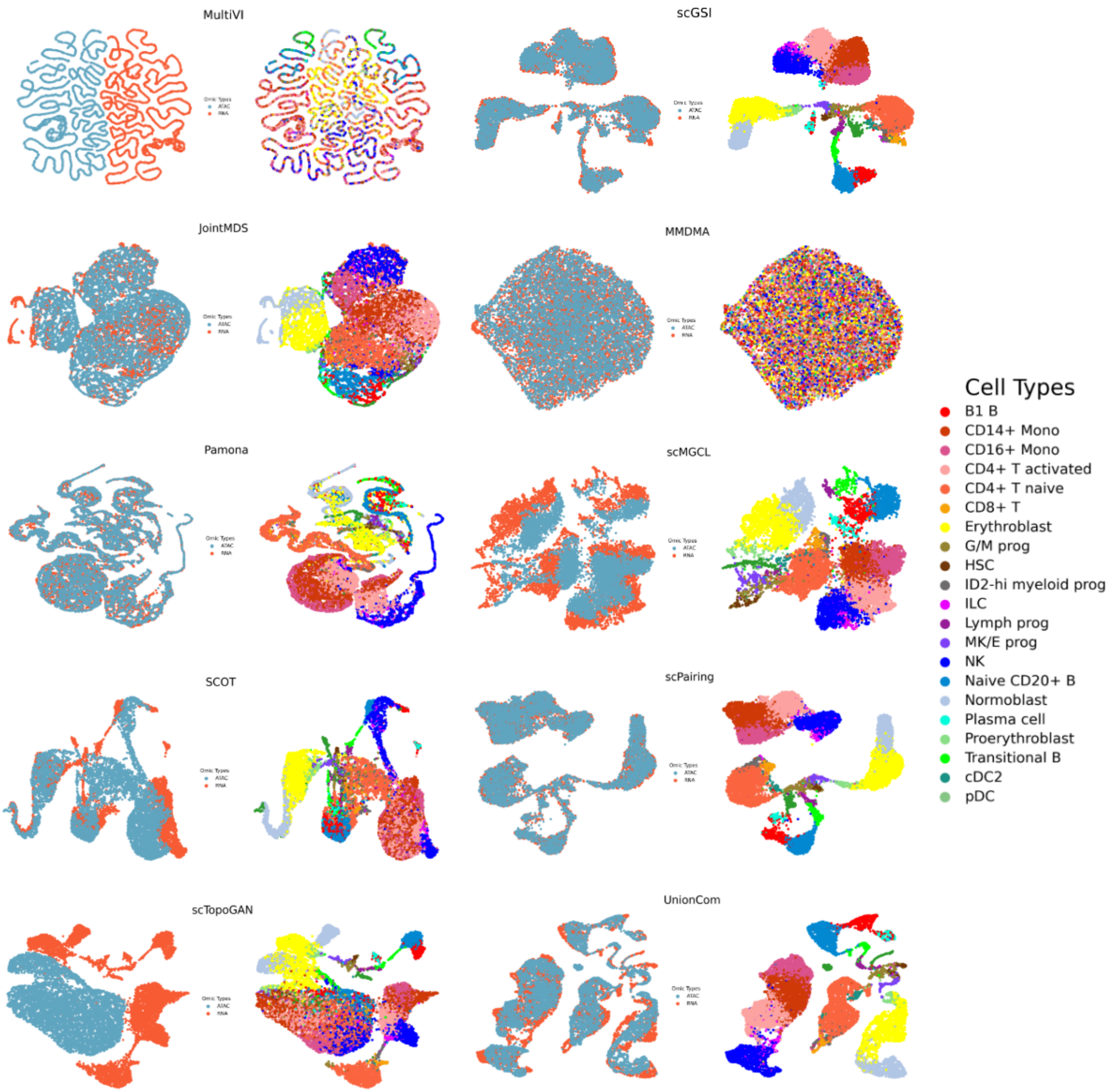

Fig A. UMAP visualizations of integration results for all methods on the BMMC dataset. The panels show, method by method, whether RNA and ATAC cells mix while major immune populations remain separated. Cells are colored by modality (left) and cell type (right).

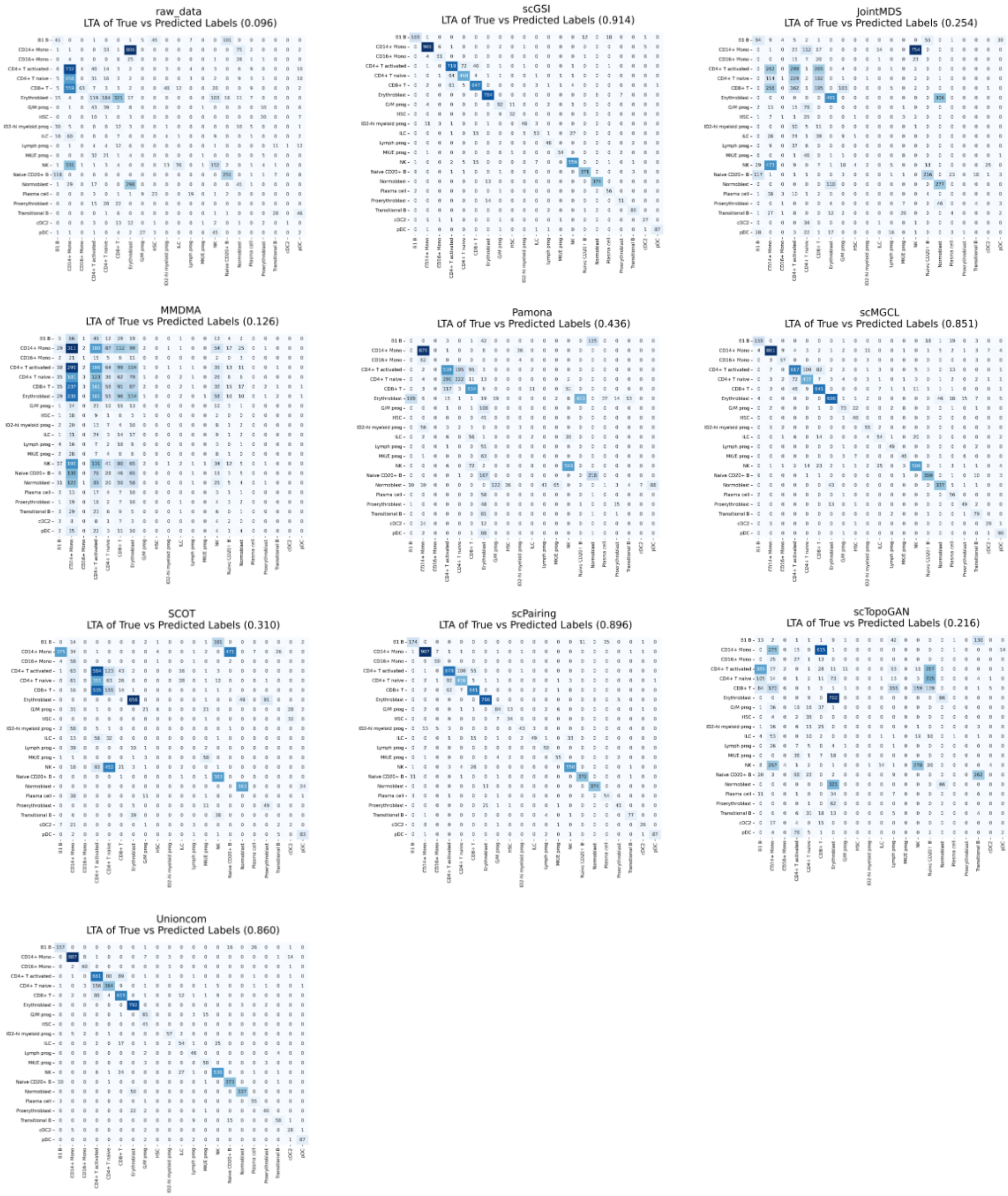

Fig B. Confusion matrices of true cell types versus predicted cell types for all methods on the BMMC dataset. The matrices show which immune subpopulations remain difficult to distinguish after ATAC-to-RNA label transfer.

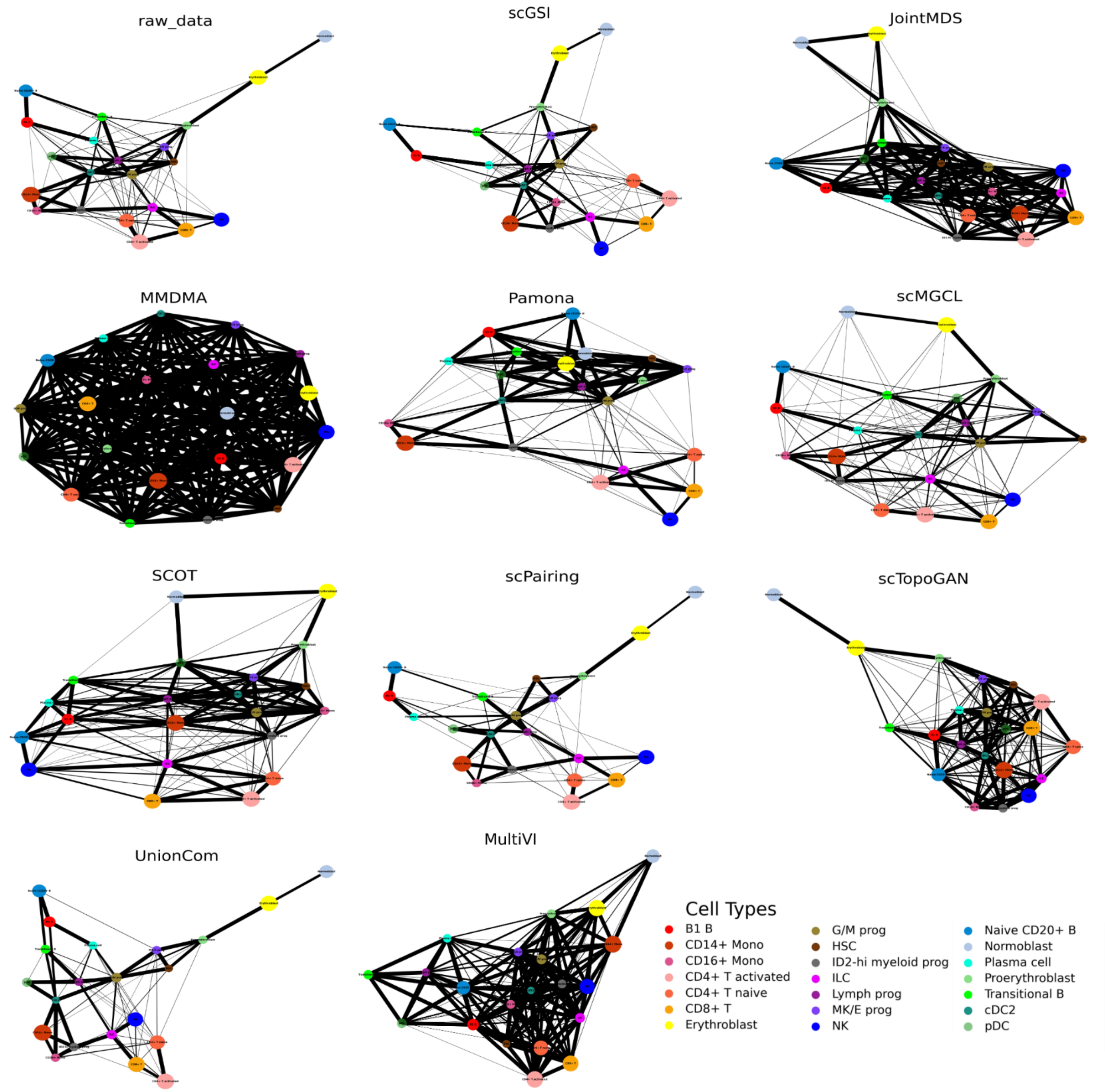

Fig C. PAGA inference visualizations for all methods on the BMMC dataset. The graphs compare how well each method preserves hematopoietic lineage organization after alignment. Each node represents a cell type, node size is proportional to the number of cells, edges denote lineage relationships, and edge thickness indicates connectivity strength.

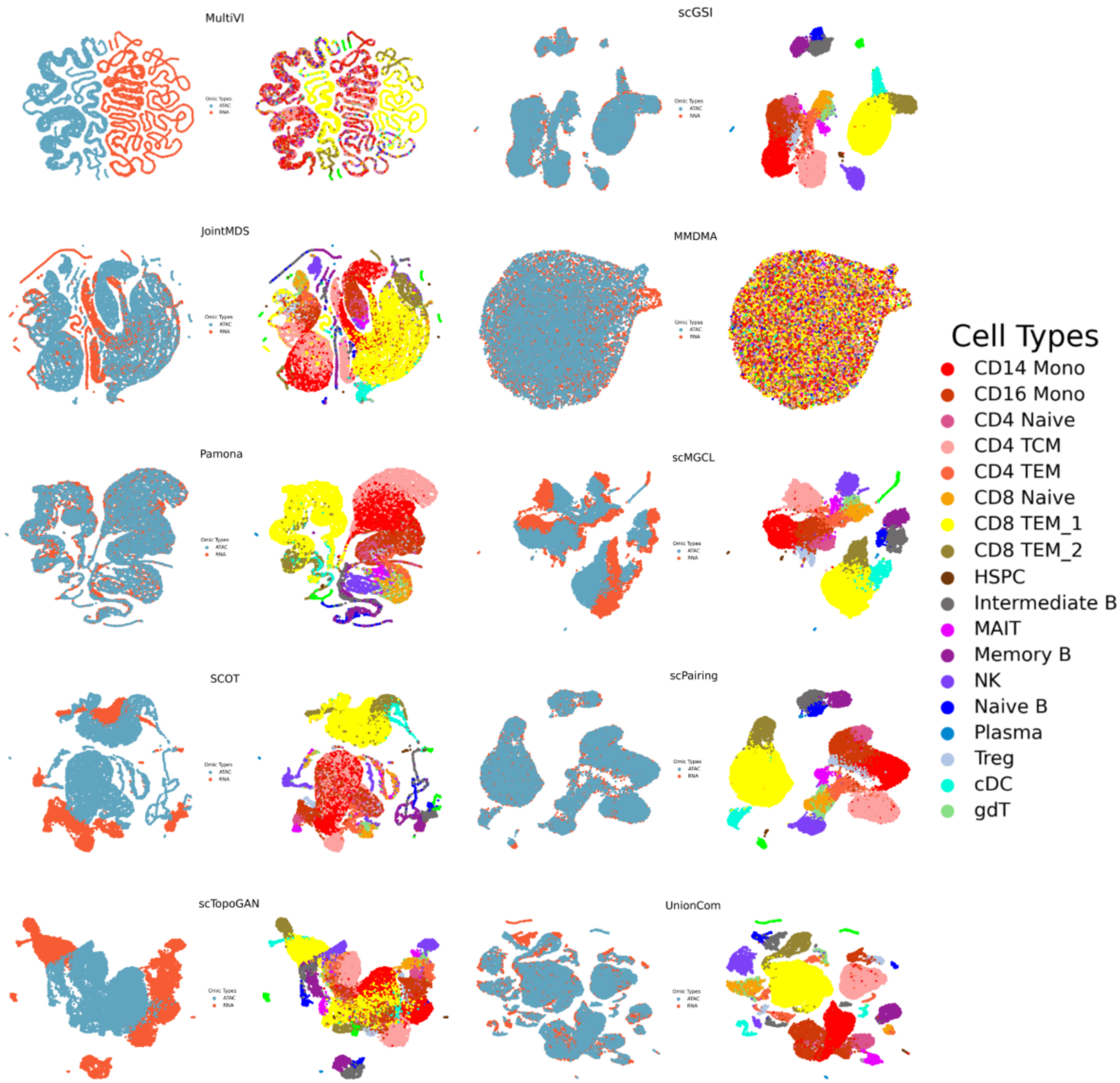

Fig D. UMAP visualizations of integration results for all methods on the CITE_PBMC dataset. These plots show whether stronger modality mixing is accompanied by loss of cell-state structure. Cells are colored by modality (left) and cell type (right).

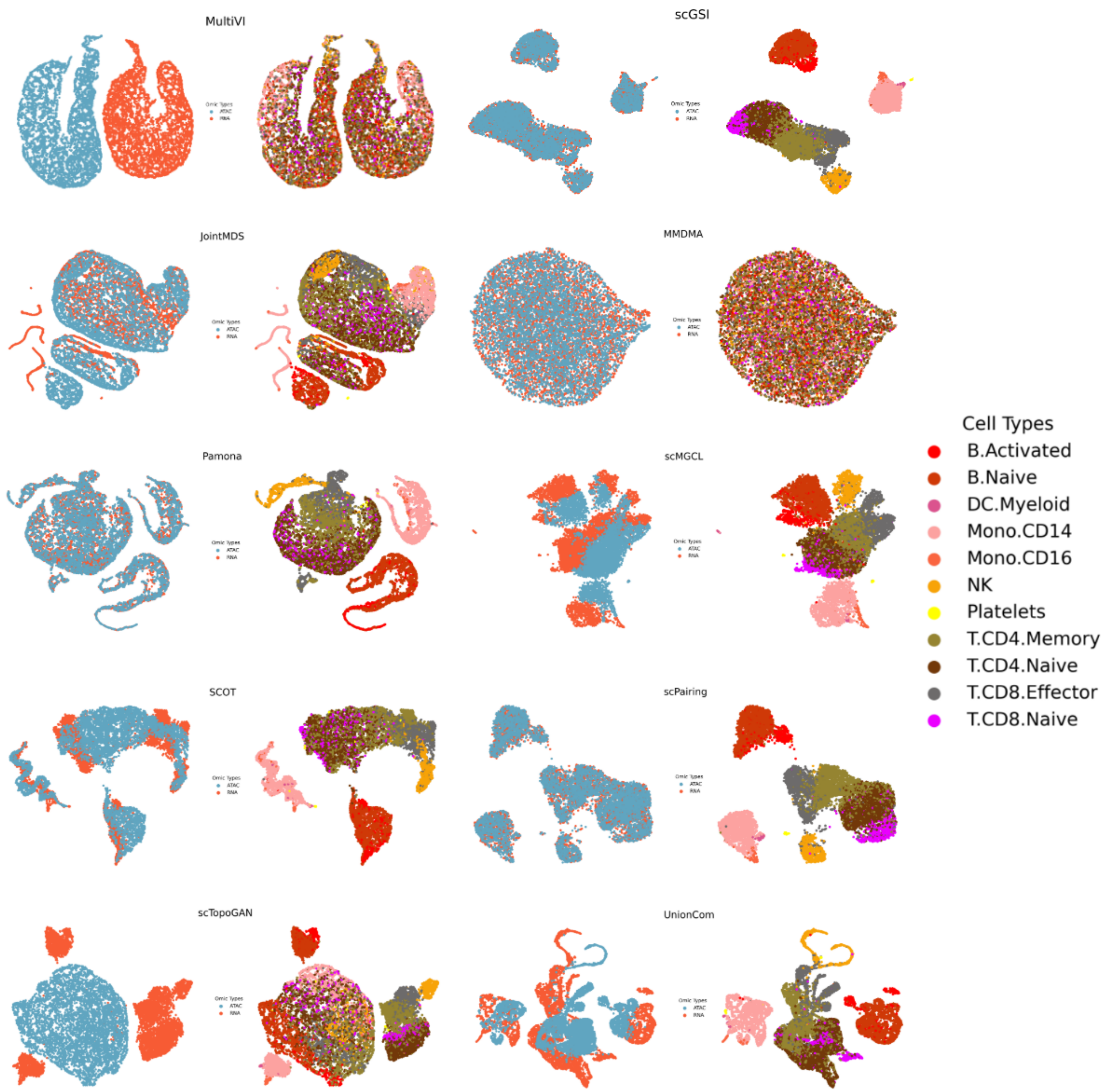

Fig E. UMAP visualizations of integration results for all methods on the TEA_PBMC dataset. The plots show how different integration methods affect subtype boundary preservation in this TEA-seq dataset. Cells are colored by modality (left) and cell type (right).

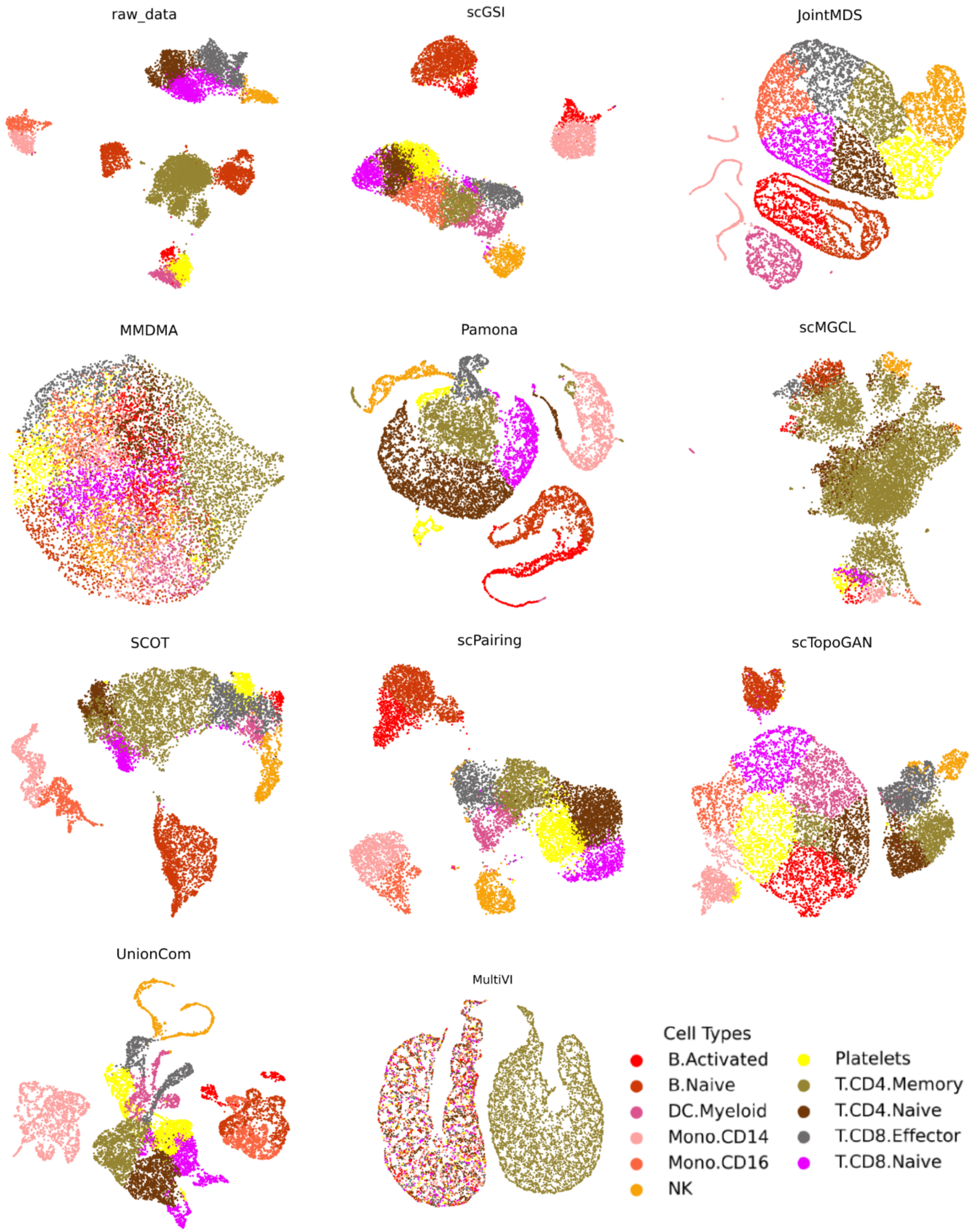

Fig F. UMAP plots of K-means clustering results for all methods on the TEA_PBMC dataset. Each panel shows how well the integrated embedding supports unsupervised cell-state partitioning after alignment. Cells are colored by cluster assignments.

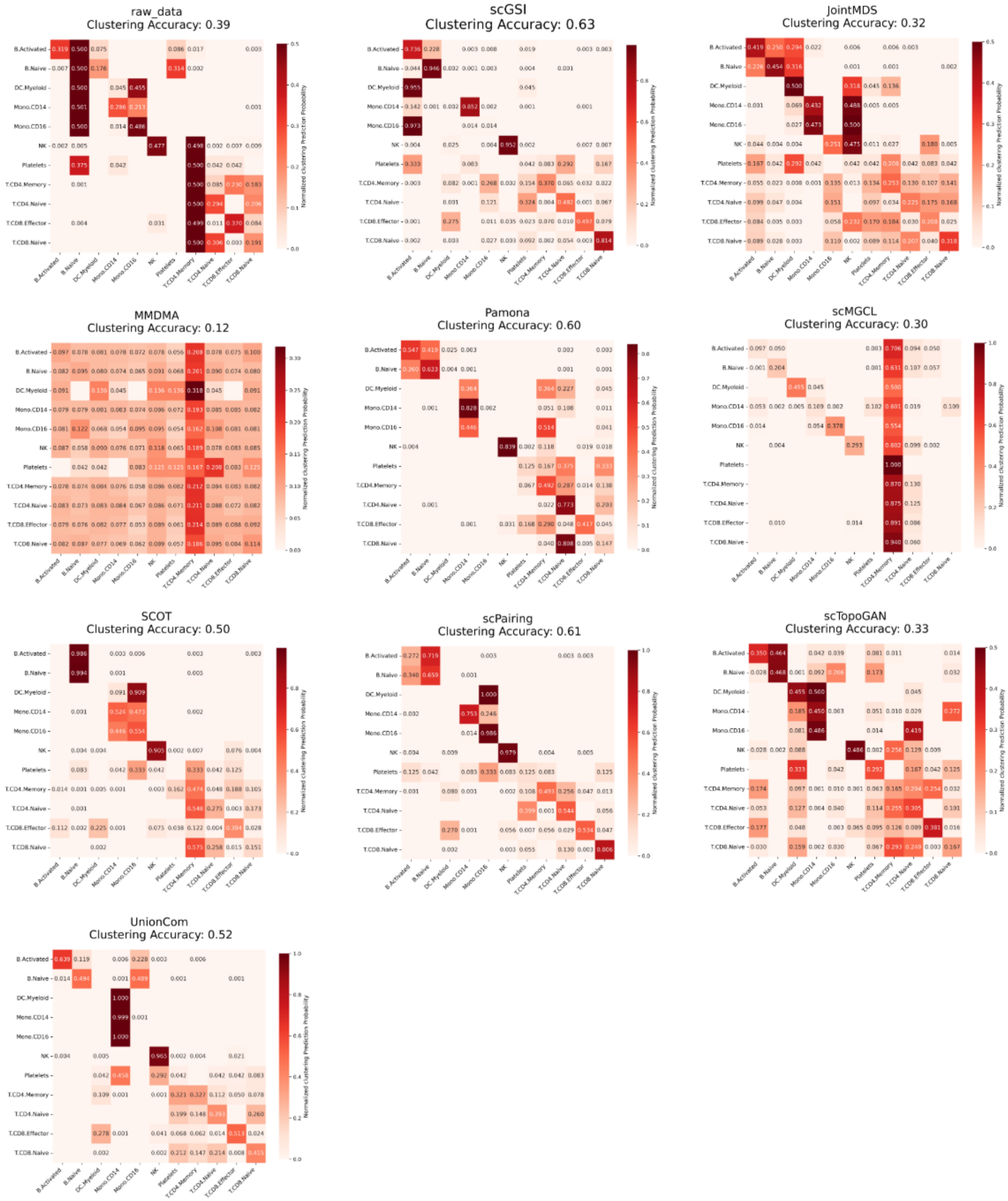

Fig G. Heatmaps of K-means clustering results for all methods on the TEA_PBMC dataset. The heatmaps show which related immune lineages remain confounded after integration. Rows represent true cell types, columns represent cluster assignments, and each element (i, j) indicates the proportion of cells of true type i assigned to cluster j.

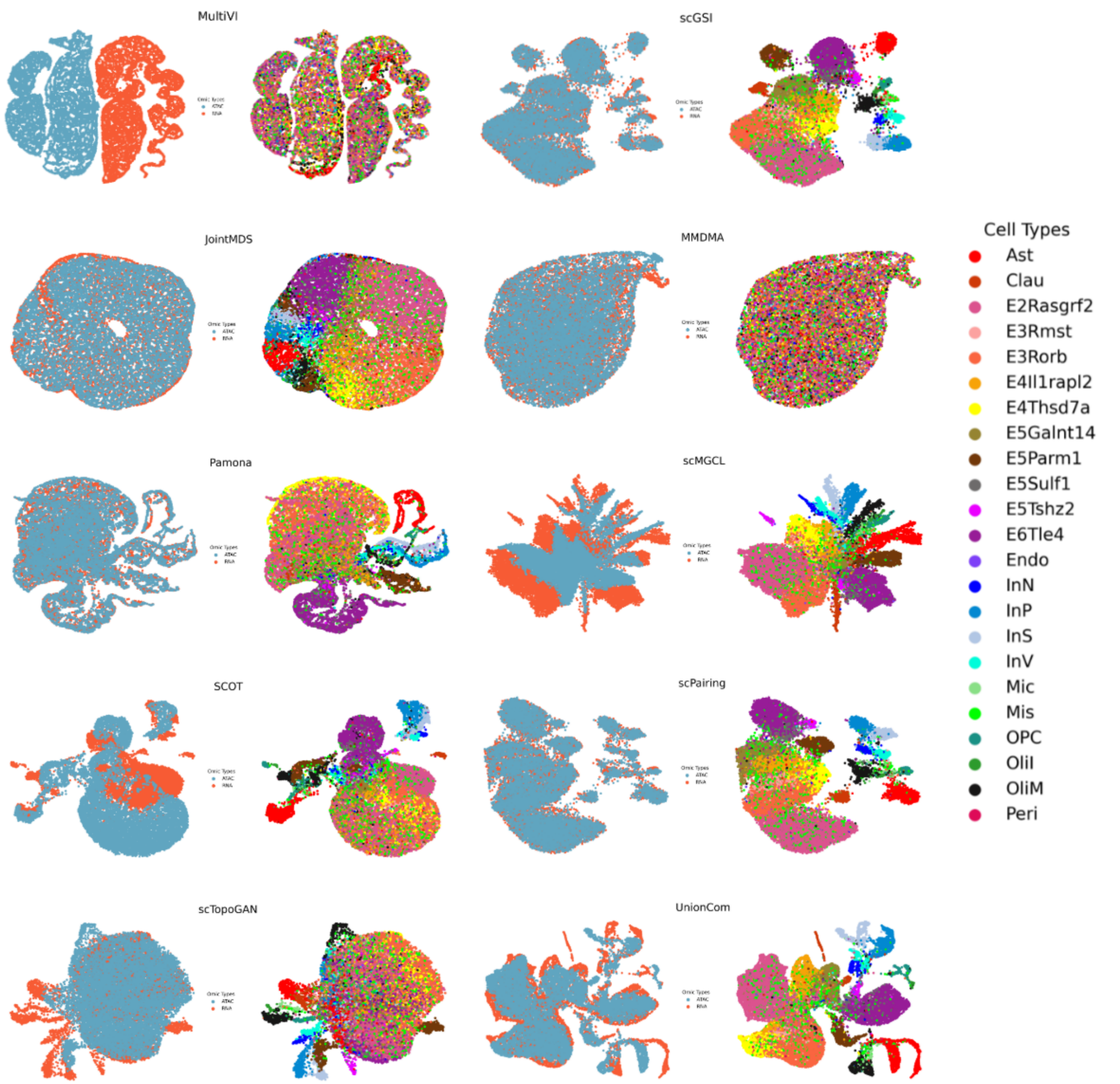

Fig H. UMAP visualizations of integration results for all methods on the AdBraCor dataset. The plots show whether aligned embeddings preserve both modality mixing and neurodevelopmentally meaningful cell-state organization in adult cortex data. Cells are colored by modality (left) and cell type (right).

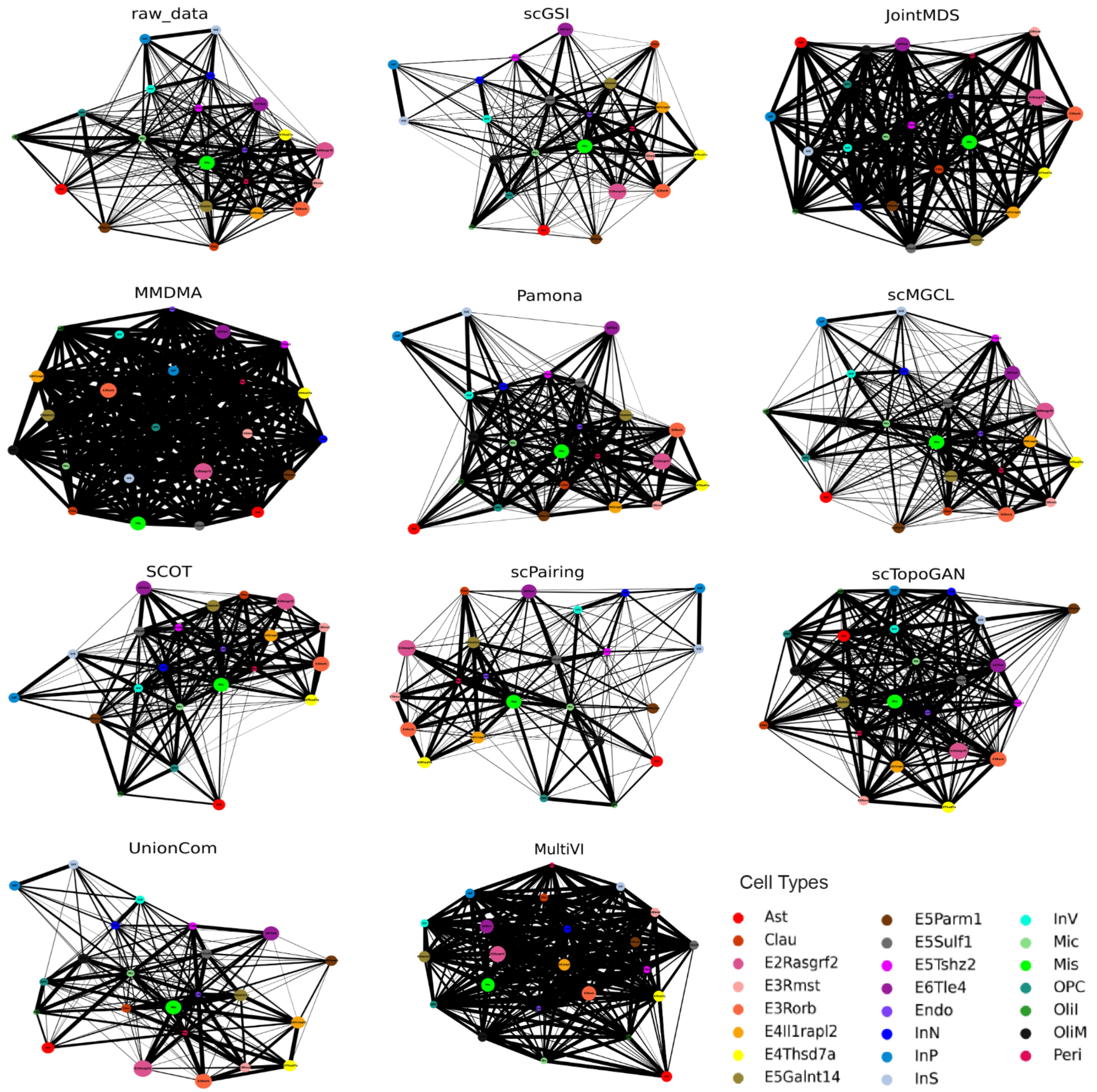

Fig I. PAGA inference visualizations for all methods on the AdBraCor dataset. The panels compare large-scale lineage topology recovered by different methods in the adult brain cortex. Each node represents a cell type, node size is proportional to the number of cells, edges denote lineage relationships, and edge thickness indicates connectivity strength.

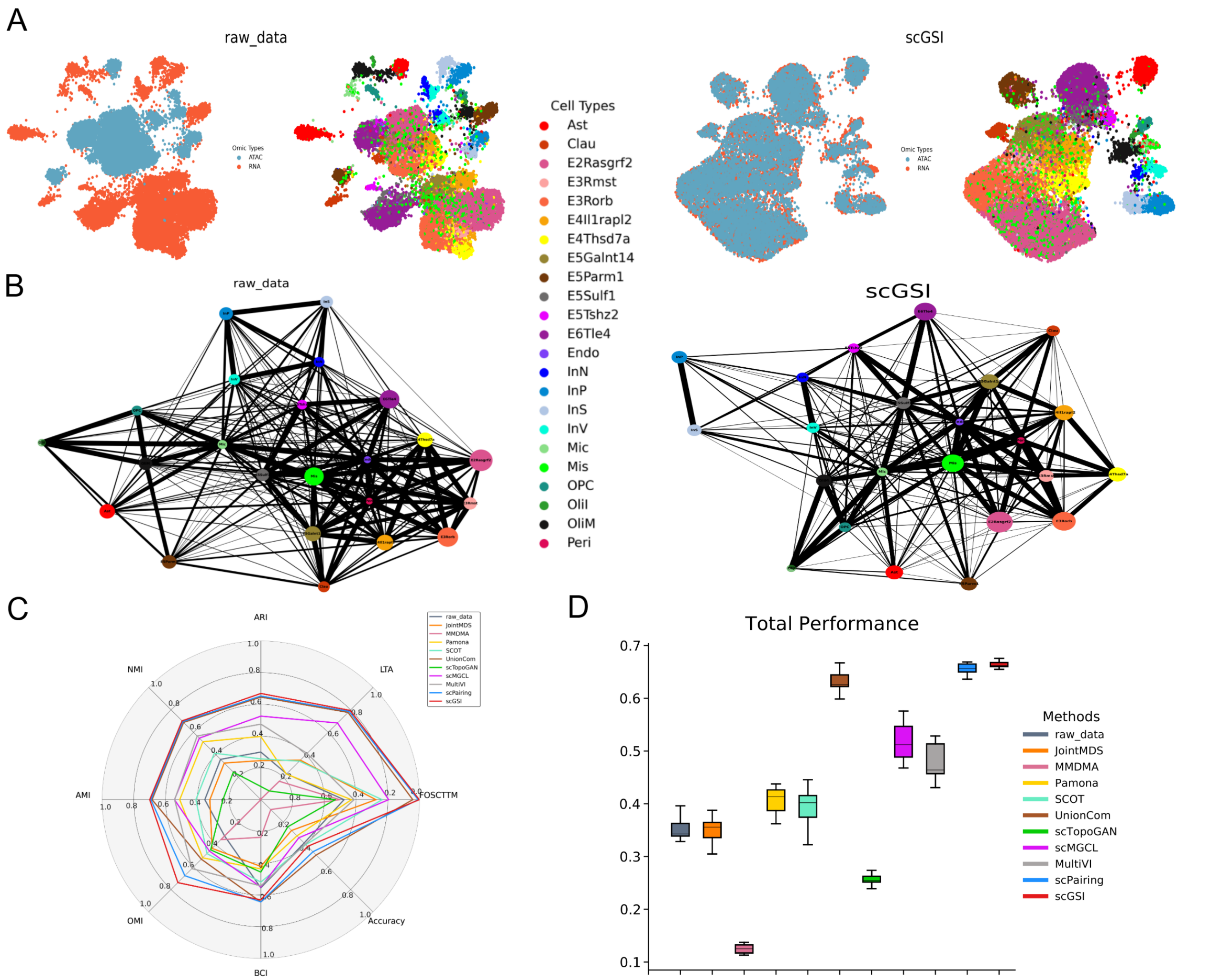

Fig J. Integration performance of scGSI on the AdBraCor dataset. The figure summarizes how scGSI behaves on adult brain cortex data after paired integration. (A) UMAP visualization of the integrated embedding. (B) PAGA trajectory inference visualization. (C) Radar plot summarizing quantitative metrics. (D) Overall performance box plot. Across the four panels, scGSI maintains strong integration quality and downstream biological interpretability on AdBraCor.

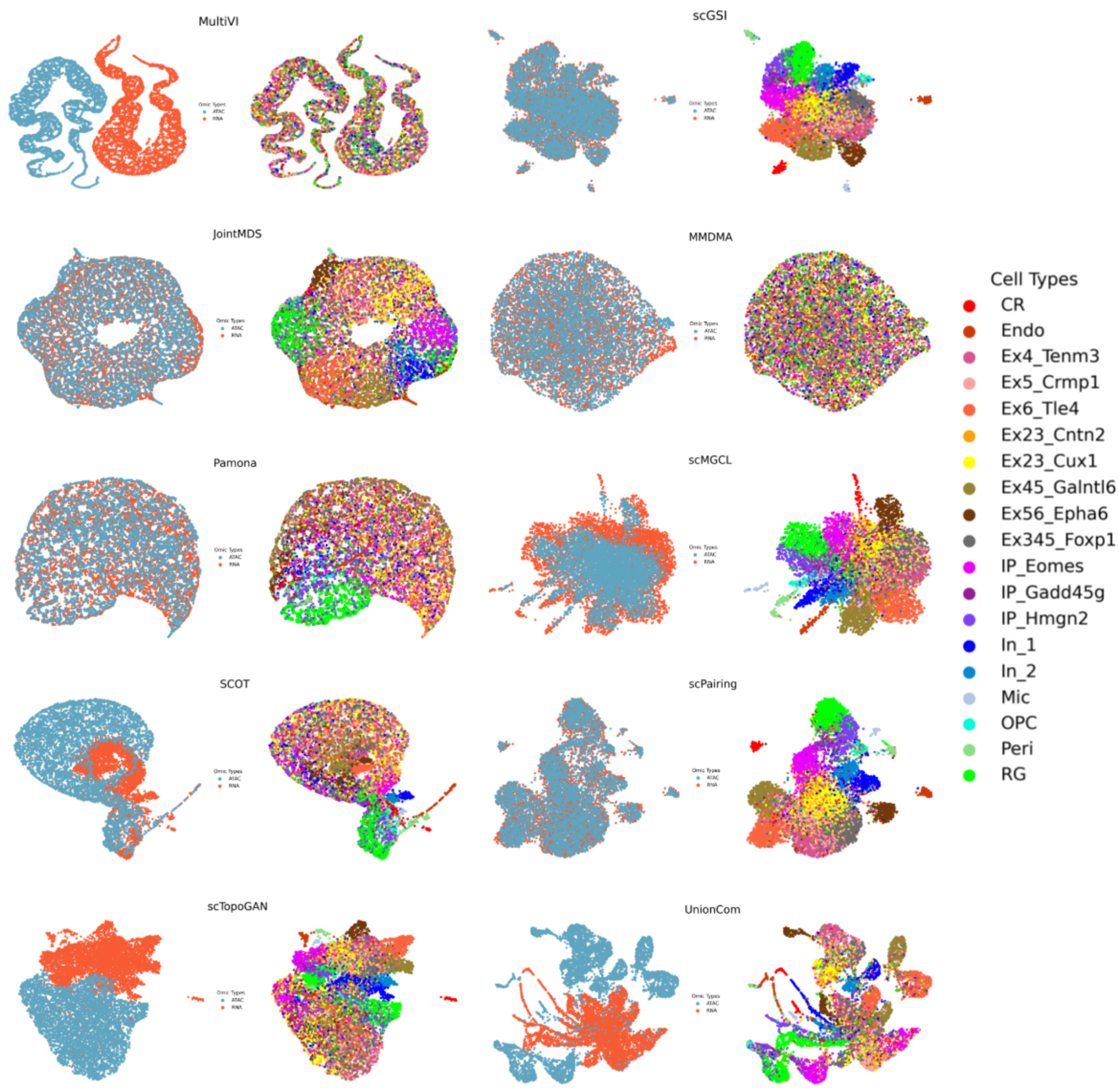

Fig K. UMAP visualizations of integration results for all methods on the P0BraCor dataset. The plots show how well each method preserves developmental cell-state organization after integration in neonatal cortex data. Cells are colored by modality (left) and cell type (right).

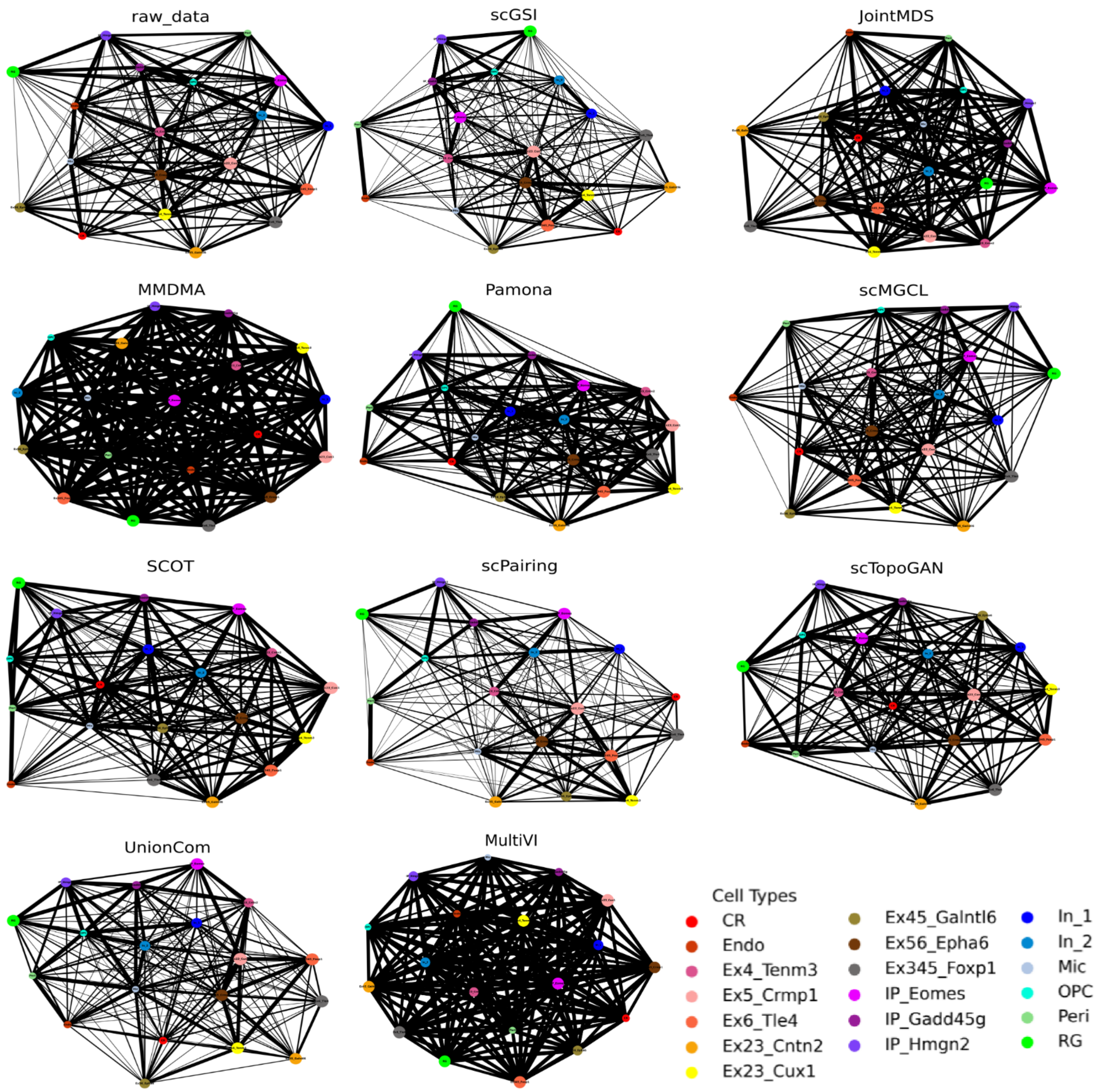

Fig L. PAGA inference visualizations for all methods on the P0BraCor dataset. The panels compare the developmental topology recovered by different integration methods in neonatal brain cortex data. Each node represents a cell type, node size is proportional to the number of cells, edges denote lineage relationships, and edge thickness indicates connectivity strength.

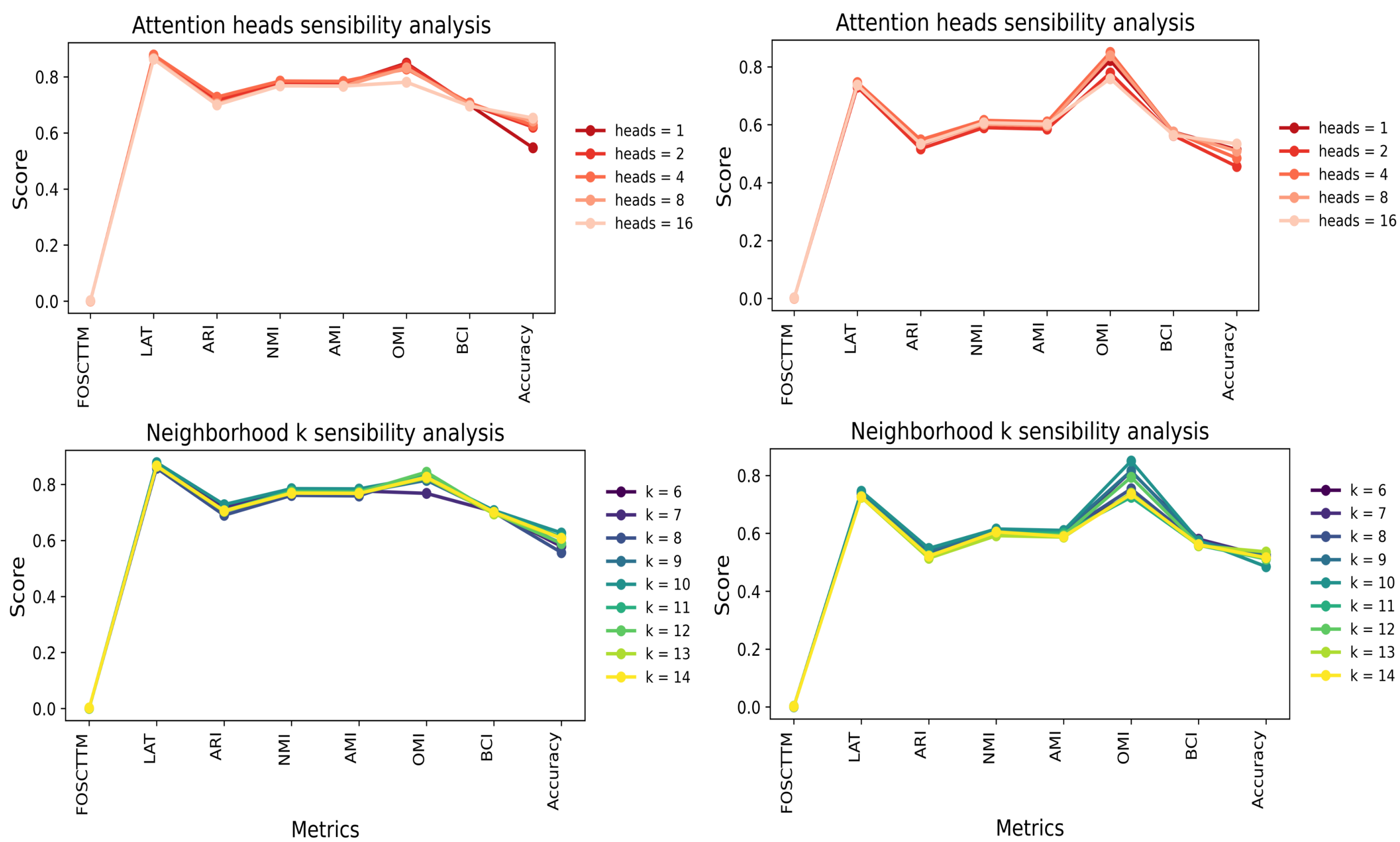

Fig M. Sensitivity of scGSI to key hyperparameters on the TEA_PBMC and P0BraCor datasets. These line graphs assess whether the performance of scGSI depends on narrowly tuned settings by varying the number of attention heads $Head$, the temperature parameter τ, and the number of nearest neighbors k.

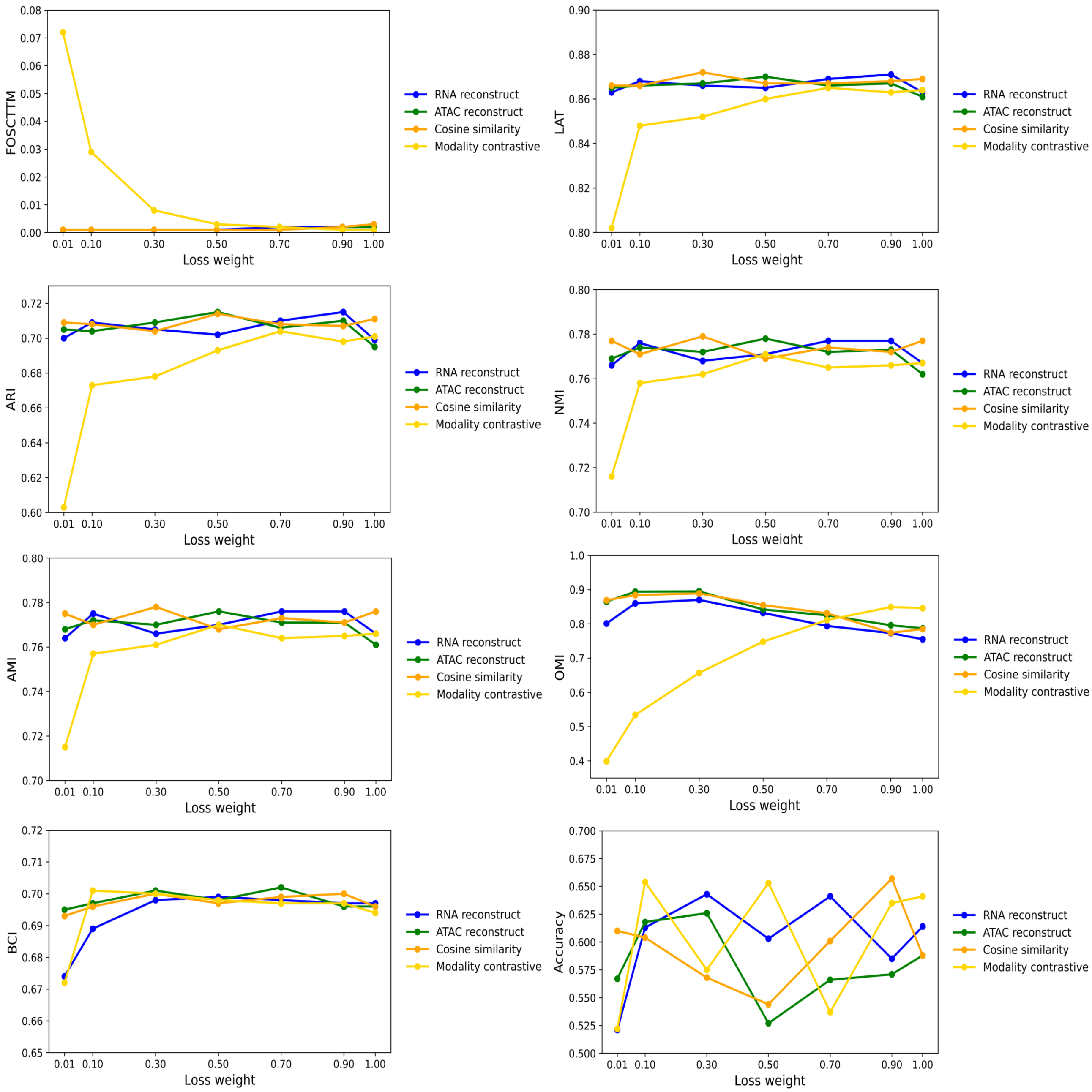

Fig N. Sensitivity of scGSI to different loss weights on the TEA_PBMC dataset. Using a univariate control strategy, the plots show which optimization terms contribute most strongly to final performance and how stable the model remains under weight perturbation.

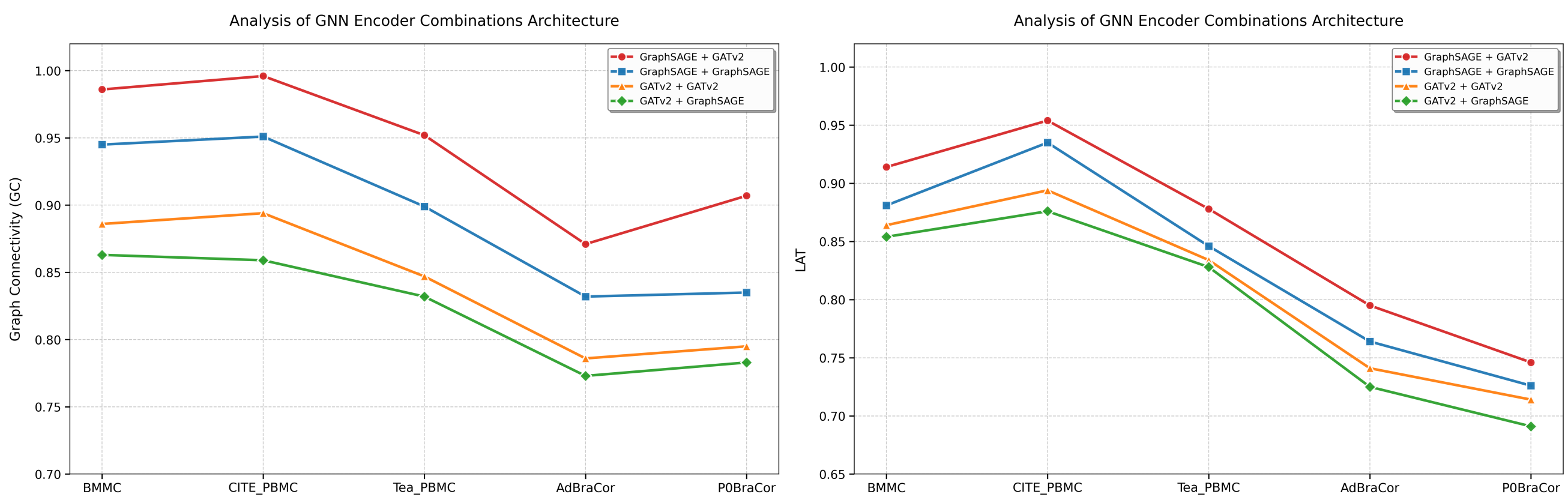

Fig O. Comparison of alternative GNN encoder combinations across datasets using multiple evaluation criteria. The figure evaluates how encoder choice influences modality-specific structure preservation and downstream alignment, with results reported for Graph Connectivity (GC) and LAT. These analyses support the use of GraphSAGE for RNA and GATv2 for ATAC in scGSI, showing that the same combination remains competitive or favorable when assessed under different objectives.

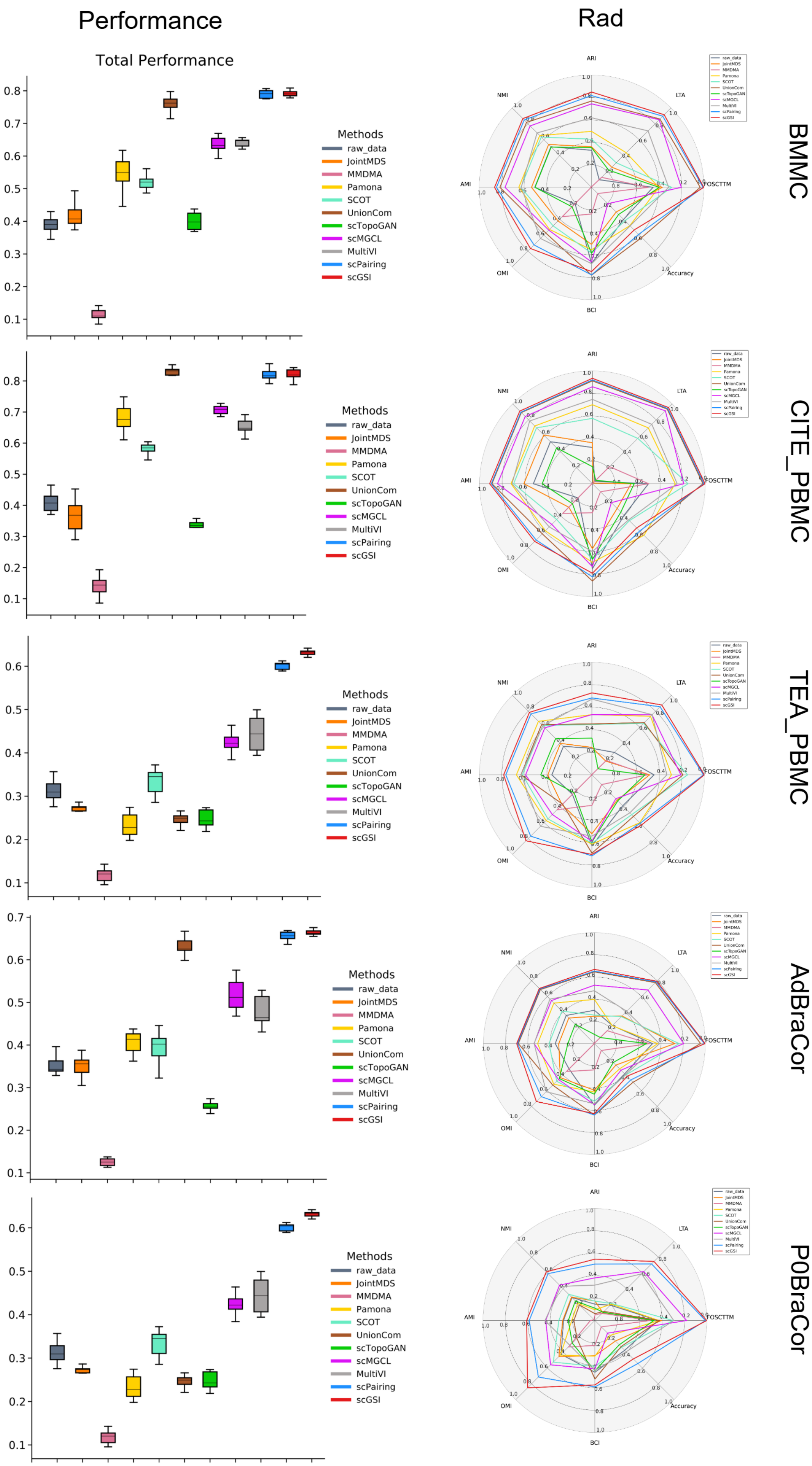

Fig P. Overall performance box plots across five benchmark datasets and dataset-specific radar plots. The left column shows overall performance scores for each method on BMMC, CITE_PBMC, TEA_PBMC, AdBraCor, and P0BraCor; the right column summarizes the corresponding per-metric overview.

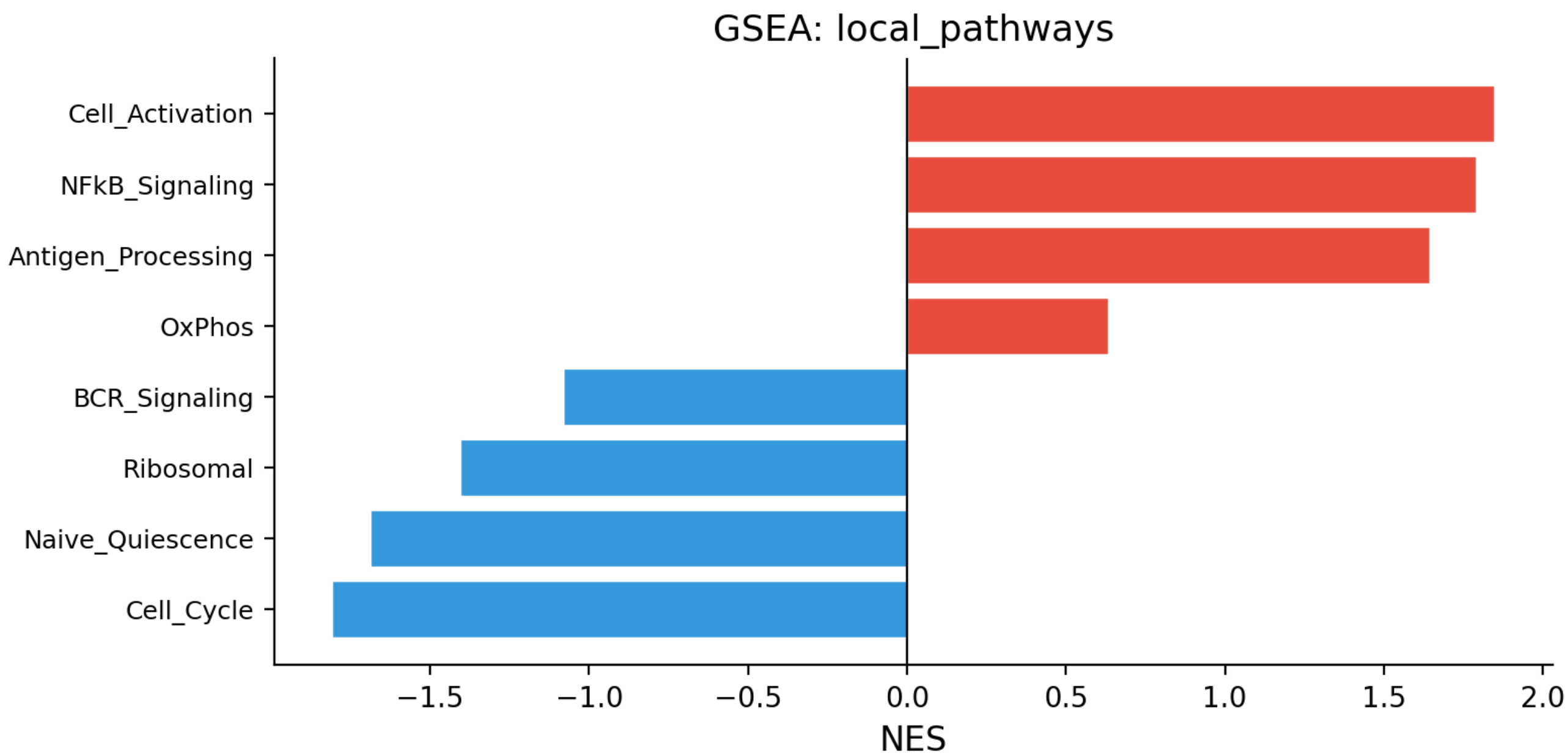

Fig Q. Differential enrichment scores of B-cell subsets across key biological pathways. Positive normalized enrichment scores indicate pathways enriched in B.Activated cells, whereas negative scores indicate pathways enriched in B.Naive cells.

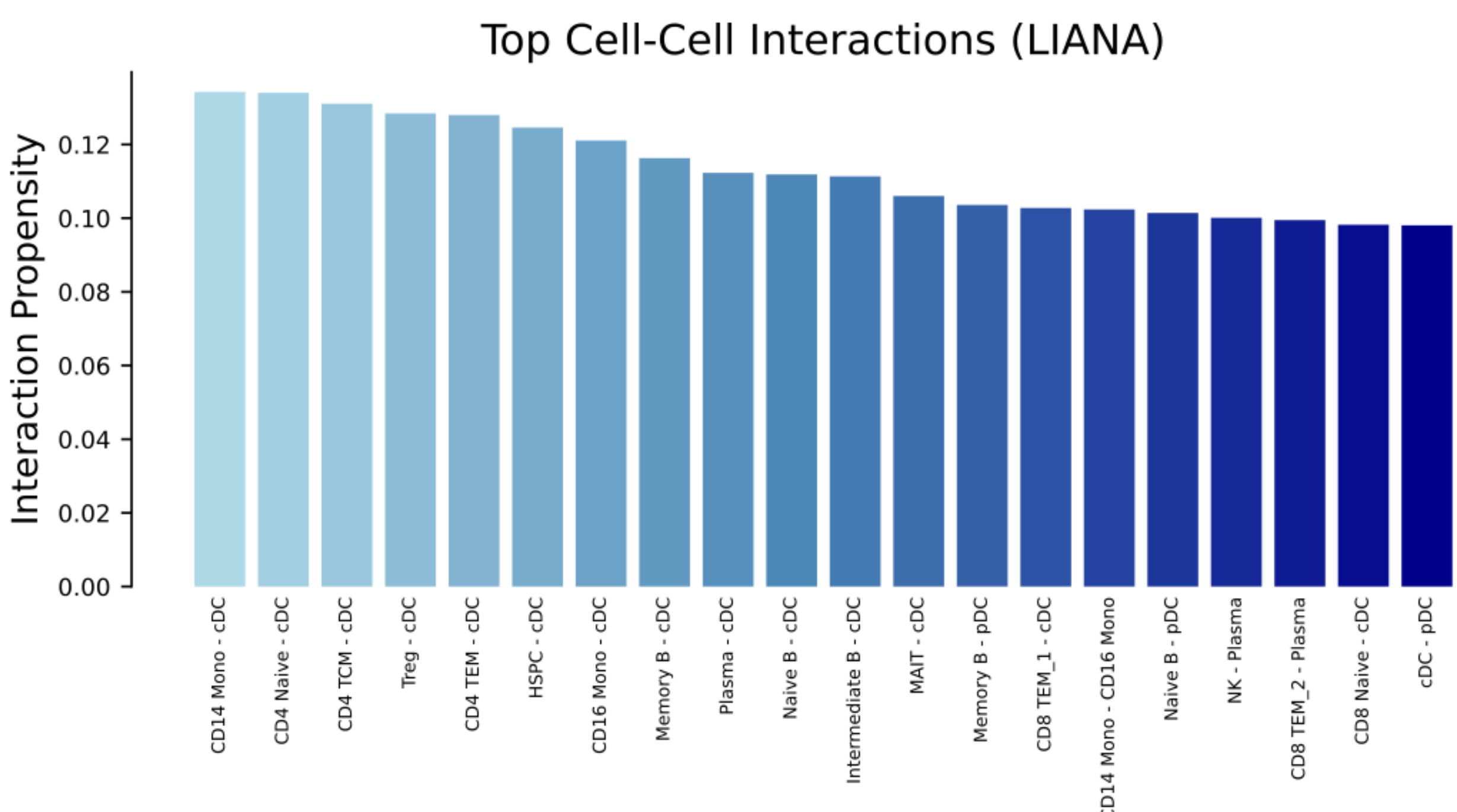

Fig R. Bar chart of the top 20 cell-type-pair interaction propensities inferred with LIANA. Each bar corresponds to a cell-type pair ranked by aggregated interaction propensity derived from LIANA ligand–receptor analysis.

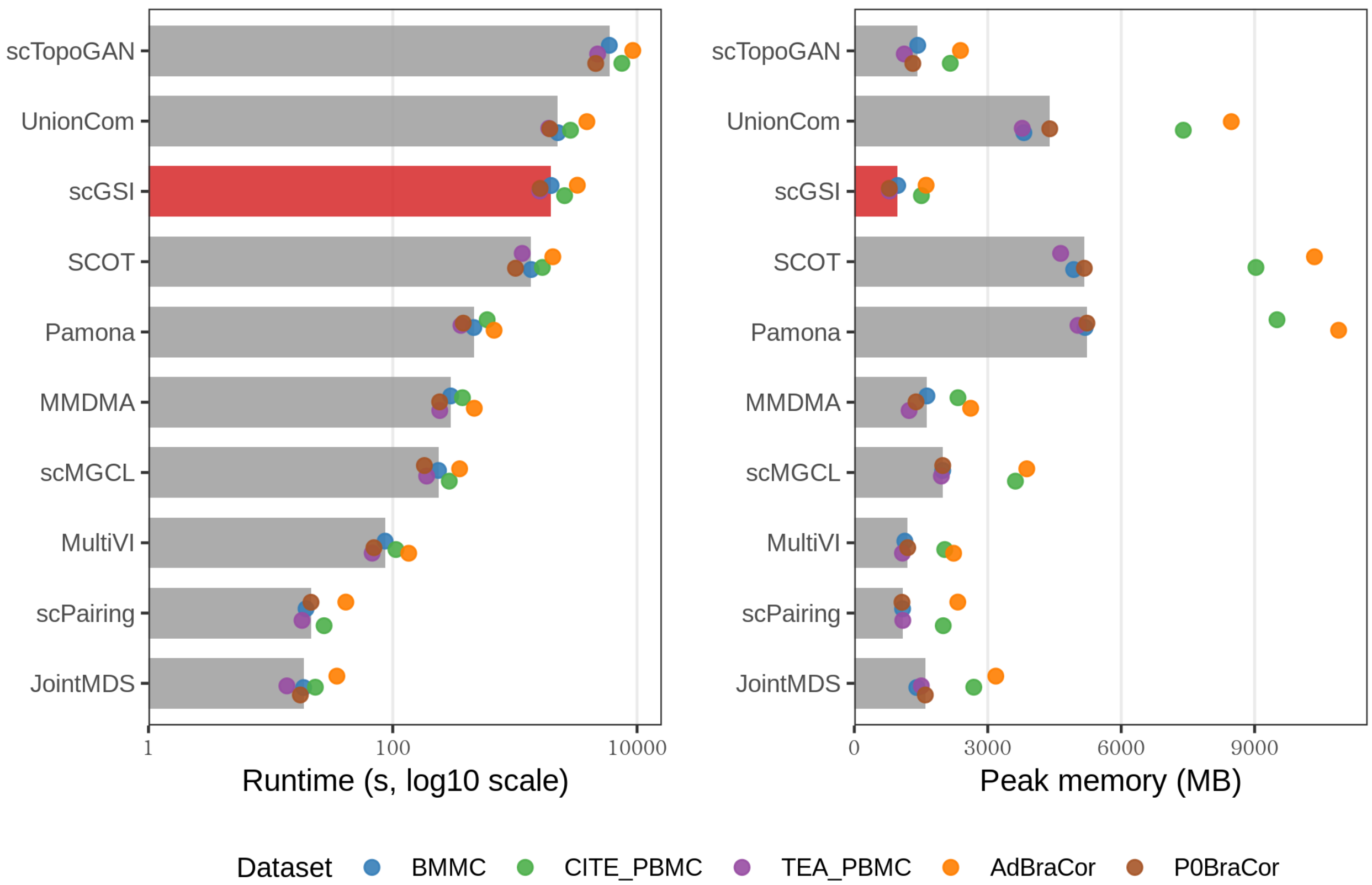

Fig S. Runtime and peak memory usage of benchmarked methods across datasets. Bars show the median value across the five datasets; colored points indicate individual dataset measurements. Methods are ordered by median runtime (left panel, $\log_{10}$ scale). scGSI is highlighted in red. All experiments were run on a workstation with an Intel Core i9-14900K CPU, 125 GB RAM, and an NVIDIA RTX 3090 (24 GB VRAM; CUDA 11.8). Runtime was measured as end-to-end wall-clock time; peak memory was recorded via `nvidia-smi` and `psutil`. Each configuration was run three times and the averages are reported.

References

[1] Joseph M Granja, M Ryan Corces, Sarah E Pierce, Denes Hnisz, Anna Shcherbina, Molly Y Hein, Andreas Mund, Myles S Brown, Ansuman T Satpathy, William J Greenleaf, et al. Archr is a scalable software package for integrative single-cell chromatin accessibility analysis. *Nature Genetics*, 53(3):403–411, 2021. https://doi.org/10.1101/2020.04.28.066498.

[2] Hannah A Pliner, Jonathan S Packer, Jonna L McFaline-Figueroa, Mark S Cembrowski, and Cole Trapnell. Cicero predicts cis-regulatory dna interactions from single-cell chromatin accessibility data. *Molecular Cell*, 71(5):858–871, 2018. https://doi.org/10.1016/j.molcel.2018.06.044.

[3] Wenbao Yu, Yasin Uzun, Qin Zhu, Changya Chen, and Kai Tan. scatac-pro: a comprehensive workbench for single-cell chromatin accessibility sequencing data. *Genome Biology*, 21(1):94, 2020. https://doi.org/10.1186/s13059-020-02008-0.

[4] Tim Stuart, Avi Srivastava, Shivani Madad, Caleb A Lareau, and Rahul Satija. Single-cell chromatin state analysis with signac. *Nature Methods*, 18(11):1333–1341, 2021. https://doi.org/10.1038/s41592-021-01282-5.

[5] Fabian A Wolf, Philipp Angerer, and Fabian J Theis. Scanpy: large-scale single-cell gene expression data analysis. *Genome Biology*, 19:15, 2018. https://doi.org/10.1186/s13059-017-1382-0.

[6] Fabian Pedregosa, Gaël Varoquaux, Alexandre Gramfort, Vincent Michel, Bertrand Thirion, Olivier Grisel, Mathieu Blondel, Peter Prettenhofer, Ron Weiss, Vincent Dubourg, et al. Scikit-learn: Machine learning in python. *Journal of Machine Learning Research*, 12:2825–2830, 2011. https://doi.org/10.3389/fninf.2014.00014.

[7] Kai Cao, Xue Bai, Xin Zhang, Yu Wang, Jun Wang, Jixuan Lu, et al. Unsupervised topological alignment for single-cell multi-omics integration. *Bioinformatics*, 36(Supplement_1):i48–i56, 2020. https://doi.org/10.1093/bioinformatics/btaa443.

[8] Akash Singh, Kajsa Biharie, Marcel JT Reinders, Ahmed Mahfouz, and Tycho Abdelaal. sctopogan: unsupervised manifold alignment of single-cell data. *Bioinformatics Advances*, 3(1):vbad171, 2023. https://doi.org/10.1101/2022.04.27.489829.

[9] Dexiong Chen, Bowen Fan, Carlos Oliver, and Karsten Borgwardt. Unsupervised manifold alignment with joint multidimensional scaling. In *The Eleventh International Conference on Learning Representations*, 2023. https://doi.org/10.48550/arXiv.2207.02968. URL `https://openreview.net/forum?id=lUpjsrKItz4`.

[10] Kai Cao, Yu Hong, and Lin Wan. Manifold alignment for heterogeneous single-cell multi-omics data integration using pamona. *Bioinformatics*, 38(1):211–219, 2021. https://doi.org/10.1101/2020.11.03.366146.

[11] Ritambhara Singh, Pinar Demetci, Giacomo Bonora, Vijay Ramani, Clarence Lee, Hongke Fang, Zhi Duan, Xinxian Deng, Jay Shendure, Christine M Disteche, and William Stafford Noble. Unsupervised manifold alignment for single-cell multi-omics data. In *Proceedings of the 11th ACM International Conference on Bioinformatics, Computational Biology and Biomedicine (BCB '20)*, pages 1–10. ACM, 2020. https://doi.org/10.1145/3388440.3412410.

[12] Pinar Demetci, Rebecca Santorella, Björn Sandstede, William Stafford Noble, and Ritambhara Singh. Scot: single-cell multiomics alignment with optimal transport. *Journal of Computational Biology*, 29(1):3–18, 2022. https://doi.org/10.1089/cmb.2021.0446.

[13] Zhenglong Cheng, Rui Lu, and Shuxiang Zhang. scmgcl: accurate and efficient integration representation of single-cell multi-omics data. *Bioinformatics*, 41(7):btaf392, 2025. https://doi.org/10.1093/bioinformatics/btaf392.

[14] Tal Ashuach, Marius I Gabitto, Michael I Jordan, and Nir Yosef. Multivi: deep generative model for the integration of multimodal data. *Nature Methods*, 20(8):1222–1231, 2023. https://doi.org/10.1038/s41592-023-01909-9.

[15] Jing Niu, Carlos Vasquez-Rios, and Jun Ding. Single-cell multiomics data integration and generation with scpairing. *Cell Reports Methods*, 2025. https://doi.org/10.1101/2025.01.04.631299. Published online October 27, 2025.
